# Targeting BCL10 by small peptides for the treatment of B cell lymphoma

**DOI:** 10.7150/thno.47533

**Published:** 2020-09-19

**Authors:** Wei Bao, Chenxia Sun, Xiaochen Sun, Miaoxia He, Haolan Yu, Wenfen Yan, Fuping Wen, Liang Zhang, Chenghua Yang

**Affiliations:** 1 Department of Urology, Changhai Hospital, Second Military Medical University, Shanghai, China, 200433.; 2 CAS Key Laboratory of Tissue Microenvironment and Tumor, Shanghai Institute of Nutrition and Health, University of Chinese Academy of Sciences, Chinese Academy of Sciences, Shanghai, China, 200031.; 3 Department of Pathology, Changhai Hospital, Second Military Medical University, Shanghai, China, 200433.; 4 Institute for Stem Cell and Regeneration, Chinese Academy of Sciences, 1 Beichen West Road, Chaoyang District, Beijing, China, 100101.; 5 Department of Plastic & Reconstructive Surgery, Shanghai Ninth People's Hospital, Shanghai Jiao Tong University School of Medicine, 639 Zhizaoju Road, Shanghai, 200011, P.R. China.

**Keywords:** Bcl10, diffuse large B-cell lymphoma (DLBCL), Bcl10 peptide inhibitors (BPIs), NF-κB, CARMA1-BCL10 supramolecular organizing centre (CB-SMOC)

## Abstract

**Rationale:** Constitutive activation of the NF-κB signalling pathway plays a pivotal role in the pathogenesis of activated B cell-like diffuse large B-cell lymphomas (ABC-DLBCLs), the most aggressive and chemoresistant form of DLBCL. In ABC-DLBCLs, the CARMA1-BCL10 (CB) complex forms a filamentous structure and functions as a supramolecular organizing centre (CB-SMOC) that is required for constitutive NF-κB activation, making it an attractive drug target for ABC-DLBCL treatment. However, a pharmaceutical approach targeting CB-SMOC has been lacking. Here, we developed Bcl10 peptide inhibitors (BPIs) that specifically target the BCL10 filamentation process.

**Methods:** Electron microscopy and immunofluorescence imaging were used to visualize the effect of the BPIs on the BCL10 filamentation process. The cytotoxicity of the tested BPIs was evaluated in DLBCL cell lines according to cell proliferation assays. Different *in vitro* experiments (pharmacokinetics, immunoprecipitation, western blotting, annexin V and PI staining) were conducted to determine the functional mechanisms of the BPIs. The *in vivo* therapeutic effect of the BPIs was examined in different xenograft DLBCL mouse models. Finally, Ki67 and TUNEL staining and histopathology analysis were used to evaluate the antineoplastic mechanisms and systemic toxicity of the BPIs.

**Results:** We showed that these BPIs can effectively disrupt the BCL10 filamentation process, destabilize BCL10 and suppress NF-κB signalling in ABC-DLBCL cells. By examining a panel of DLBCL cell lines, we found that these BPIs selectively repressed the growth of CB-SMOC-dependent DLBCL cells by inducing apoptosis and cell cycle arrest. Moreover, by converting the BPIs to acquire a D-retro inverso (DRI) configuration, we developed DRI-BPIs with significantly improved intracellular stability and unimpaired BPI activity. These DRI-BPIs selectively repressed the growth of CB-SMOC-dependent DLBCL tumors in mouse xenograft models without eliciting discernible adverse effects.

**Conclusion:** We developed novel BPIs to target the BCL10 filamentation process and demonstrated that targeting BCL10 by BPIs is a potentially safe and effective pharmaceutical approach for the treatment of ABC-DLBCL and other CB-SMOC-dependent malignancies.

## Introduction

Activated B cell-like diffuse large B cell lymphoma (ABC-DLBCL) is the most aggressive subtype of DLBCL, with a five-year progression-free survival rate of approximately 40-48% 1, 2. It responds poorly to standard R-CHOP immunochemotherapy 1. No FDA-approved targeted drugs are currently available. Activating mutations and translocations of genes in B cell receptor (BCR) and Toll-like receptor (TLR) signalling pathways are frequently found in ABC DLBCL. These modified genes usually lead to the constitutive activation of the NF-κB pathway, which is a key factor in the pathogenesis of ABC-DLBCL 3-5. The addiction to constitutive NF-κB activation by ABC-DLBCL cells makes NF-κB an attractive target for therapeutic intervention. However, NF-κB is a major transcription factor with broad biological importance in inflammation and immunity regulation, making it difficult to block without causing serious side effects. For therapeutic purposes, it is therefore more desirable to target specific events upstream of NF-κB activation in ABC DLBCL cells.

In BCR-induced NF-κB activation, a protein complex consisting of CARMA1, BCL10 and MALT1 (the CBM complex) plays a key role in mediating NF-κB activation. When activated, CARMA1 nucleates BCL10 to form a filamentous high-order structure, the CB (CARMA1-BCL10) complex, which serves as a supramolecular organization centre (CB-SMOC) that drives NF-κB activation. CB-SMOC mediates NF-κB activation in two different ways. First, CB-SMOC provides a central platform that recruits and organizes NF-κB downstream signalling molecules, including MALT1, cIAP1/2, TRAF6, and the IKK complex into its high-order structure, which promotes proximity-mediated intermolecular interactions and activation 6-10; second, CB-SMOC recruits and activates MALT1 7, 10, 11, which promotes NF-κB activation by cleaving and inactivating its negative regulators A20 and CYLD 12, 13. It has been demonstrated that supramolecular organizing centre formation is a common mechanism involved in many signal transduction processes 14-17.

BCL10 filament formation is a key step in the formation of CB-SMOC 9, 11, 18, 19. BCL10 is composed of an N-terminal CARD domain and a C-terminal serine/threonine-rich domain. BCL10 self-association and filamentation rely on its CARD domain via a CARD-CARD interaction. A single mutation in the BCL10 CARD domain effectively disrupts its self-association and dominant negatively inhibits NF-κB activation 9, suggesting that the interface of BCL10 self-association is confined and can be pharmacologically manipulated. Importantly, the depletion of BCL10 in mice or humans is not lethal but leads to defects in immune responses 20-25. In addition, targeting the CBM complex can also prime tumors for successful immune checkpoint therapy 26. Given these findings, we reasoned that the filamentation process of BCL10 may represent an effective and relatively safe target for the pharmaceutical inhibition of CB-SMOC formation in B cell lymphomas.

Here, we develop novel BCL10 peptide inhibitors (BPIs) that not only can inhibit intracellular BCL10 polymerization but can also destabilize the BCL10 protein in lymphoma cells. We show that these BPIs can effectively suppress NF-κB activation and inhibit ABC-DLBCL cell proliferation. By examining a panel of DLBCL cell lines, we found that these BPIs effectively and selectively repress the growth of CB-SMOC-dependent DLBCL cells by inducing apoptosis and cell cycle arrest. By converting the BPIs to acquire a D-retro inverso (DRI) configuration, we generated DRI-BPIs with significantly improved intracellular stability and unimpaired BPI activity. We demonstrated that these DRI-BPIs can effectively inhibit ABC-DLBCL cell growth not only *in vitro* but also in mouse xenograft models, without eliciting discernible adverse effects to the mice. Therefore, our study indicated that the inhibition of the BCL10 polymerization process by peptide inhibitors is a potentially safe and effective approach for the treatment of ABC-DLBCLs.

## Materials and Methods

### Peptides

Designed BCL10 inhibitor peptides were synthesized by ChinaPeptides Co., Ltd. (Shanghai, China) and stored lyophilized at -80 °C and reconstituted with DMSO immediately before use. The purity of these peptides was 95% or higher as determined by HPLC-MS.

### Cell lines

The DLBCL cell lines were adopted from the Ari Melnick laboratory (May 2012) and cultured as previously described 27. Briefly, HBL1, TMD8, RCK8, FARAGE, U2932, SU-DHL4, SU-DHL6, Karpas-422, and MD901 cell lines were cultured in RPMI 1640 medium (Invitrogen, CAT. NO. 22400105) +10% FBS (Gibco, Cat. No. 10091148) +pen/strep (Invitrogen, Cat. No. 15140122); OCI-Ly3 was grown in RPMI 1640 medium+20% FBS+pen/strep; and OCI-Ly1 and OCI-Ly7 cells were grown in IMDM (Invitrogen, Cat. No. 12440053) +10% FBS +pen/strep. All cell lines used in the experiments were maintained at 37 °C with 5% CO_2_ in a humidified environment. The identities of all the cell lines were confirmed via short tandem repeat (STR) profiling. All cell lines were examined to ensure that they were free of mycoplasma.

### Electron microscopy

MBP-BCL10 was expressed and purified as previously described 9. MBP-BCL10 and BCL10 peptide inhibitors were premixed at a 1:1 or 1:2 molar ratio at RT for 30 minutes before adding TEV to cleave the MBP tag. 5 μL of reaction mixture was applied to copper grids (Beijing Zhongjingkeyi Technology Co., Cat. No. BZ10024a), where it remained for 1 minute and was then negatively stained with 3% uranyl acetate for 1 minute, air-dried and imaged with a JEM-1230 TEM at 100 keV.

### Immunofluorescence

A total of 5000 HeLa cells were seeded in a 12-well plate and cultured in DMEM+10% FBS. The cells were transfected with 1 μg of pcDNA4-myc-Bcl10 vector for 24 h and then treated with DMSO or BPIs (100 μM). Twenty-four hours later, the cells were fixed with 4% paraformaldehyde and permeabilized with 0.1% Triton X-100 in PBS. Cellular Bcl10 was detected with immunoblotting by Bcl10 antibody (Santa Cruz, Cat No. sc-5611) and secondary antibody (Bioworld, Cat. No. BS10029) and visualized with a Leica fluorescence microscope. Cells containing Bcl10 filaments were counted in over 10 HPF, and the percentage of cells containing Bcl10 filaments was calculated and plotted.

### Immunoblotting

Cell pellets were lysed in RIPA buffer (50 mM Tris at pH 8.0, 150 mM NaCl, 1% NP-40, 1 mM EDTA, and 1X protease inhibitor cocktail) or RIPA buffer containing 0.1% SDS. Equal amounts of protein extracts were separated by SDS-PAGE and blotted onto polyvinylidene difluoride (PVDF) membranes. The following Antibodies were purchased and used in this study: anti-IκBα (10268-1), anti-IKKβ (20979-1), anti-Bcl2 (12789-1-AP), anti-GAPDH (10494-1-AP), and anti-β-actin (66009-1-AP) were from Proteintech; anti-p-IκBα (2859S), anti-p-IKKβ (2697S), anti-RelB (10544), anti-α-tubulin (5335), anti-PARP (9532S), and anti-CARMA1 (4435) were from Cell Signaling Technology; anti-β-actin (A1978) was from Sigma; anti-Bcl10 (A1106) was from ABclonal; anti-A20 (sc-166692), anti-CYLD (sc-74435), and anti-MALT1 (sc-46677) were from Santa Cruz; anti-HOIL-1 (HPA024185) was from Atlas Antibodies; and anti-Myc (ab9106) was from Abcam.

### Growth inhibition determination

A CellTiter-Glo luminescent cell viability assay (Promega, Cat. No. G7573) was used to assess cell viability following procedures described in the product instructions. Briefly, 1 × 10^4^ exponentially growing DLBCL cells in 100 µL of medium were seeded into black 96-well microtiter plates (Corning, Cat. No. 3603) and incubated in medium containing either vehicle control (DMSO) or Bcl10 peptides (in 2-fold serial dilutions starting at 100 μM) for 24-72 h at 37 °C in 3 replicates on the same plate. After 24-72 h of incubation, 100 µL of CellTiter-Glo reagent was added to each well, mixed and then incubated for an additional 1 h at 37 °C. The luminescence signal in each well was measured in a Molecular Devices SpectraMax Paradigm instrument. The percentage of cell growth inhibition was calculated by comparing luminescence readings obtained from treated and control cells, accounting for the initial cell population (time 0). GraphPad Prism software was used to determine GI50 values.

### Statistics

The quantitative data are presented as the means ± SD. Statistical significance is reported in the figures and in the Figure legends. One-way ANOVA with Dunnett's test was used to compare values among different experimental groups using the GraphPad 6 program. For experiments with only two groups, Student's t-test (or Mann-Whitney U test) was used as specified in the Figure legends. *p* < 0.05 was considered statistically significant. *p < 0.05, **p < 0.01, ***p < 0.001, ****p < 0.0001; ns is not significant.

## Results

### Design of peptide inhibitors targeting the BCL10 self-association interface

**Figure 1:**
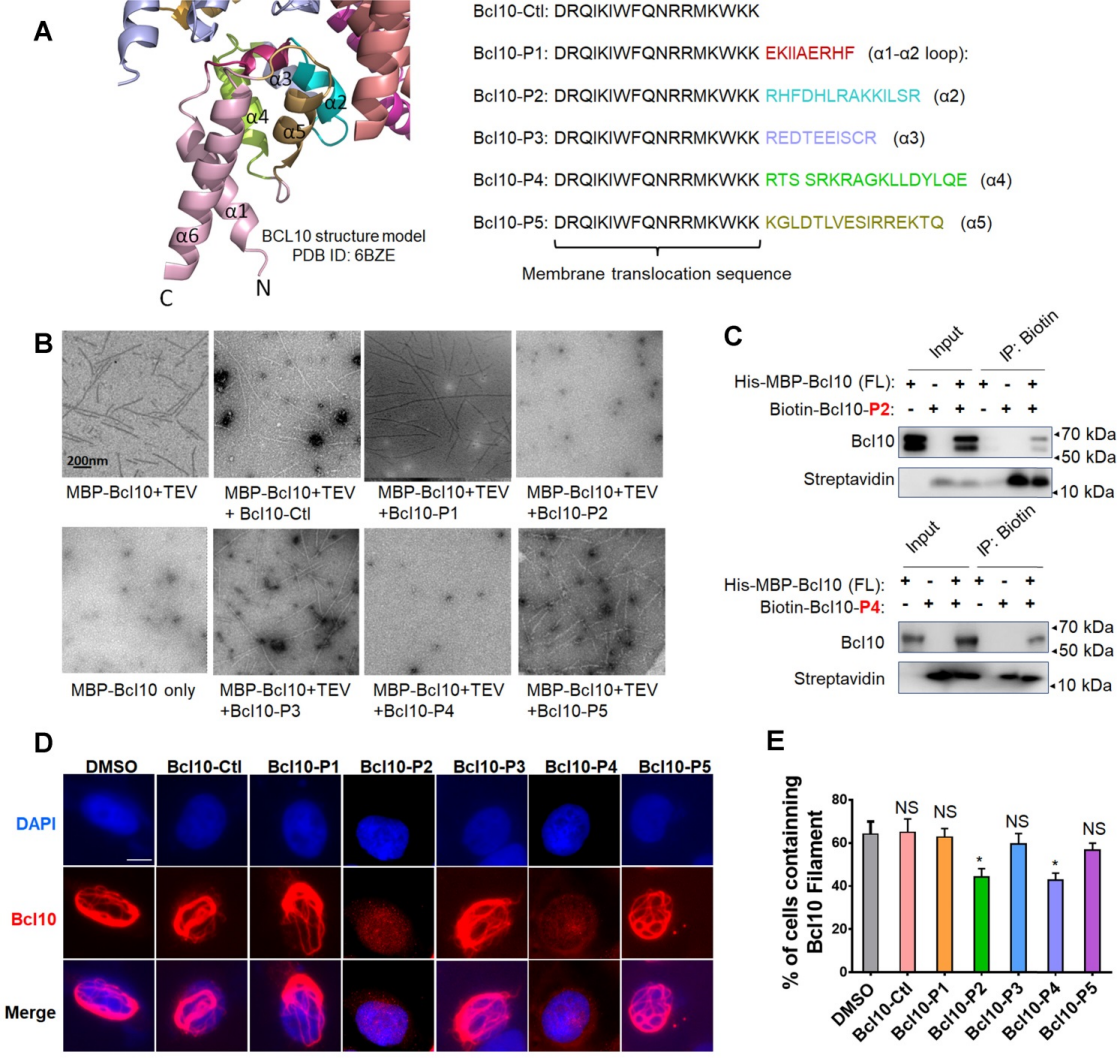
** Rationally designed BCL10 peptide inhibitors (BPIs) inhibited BCL10 filament formation. A**. BCL10 peptide inhibitor candidates were designed according to the BCL10 structure model (PDB ID: 6BZE). The sequences of BPIs are shown in the same colours as their counterparts. BCL10-Ctl consists of only the membrane translocation (MT) portion.** B**. Negative-stained EM micrographs showed the effect of different BPI candidates on BCL10 filament formation. BCL10-P2 and BCL10-P4 effectively inhibited BCL10 filament formation. **C**. BCL10-P2 and BCL10-P4 coimmunoprecipitated with BCL10. **D-E**. HeLa cells were transfected with 1 µg of pcDNA4-myc-Bcl10 vector and, after 24 h, were treated with the indicated BPIs (100 µM) for 24 h. Representative immunofluorescence images show the effect of BPIs on BCL10 filament formation in HeLa cells. **E**. Cells containing BCL10 filaments were counted in over 10 HPF. The percentage of cells containing BCL10 filaments was calculated and is shown in E. The corresponding statistics were calculated. The data are reported as the mean ± SD of three independent experiments. Statistics: one-way ANOVA with Dunnett's multiple comparison test, *p < 0.05.

BCL10 filamentation is a key step in CB-SMOC formation and NF-κB activation in ABC-DLBCL cells. The filamentation process of BCL10 relies on its CARD domain via CARD-CARD interactions 9, 10. We have previously reported that a single mutation (e.g., E53R) in the CARD domain can effectively disrupt the CARD-CARD interaction and cause dominant negative (DN) inhibition of the BCL10 filamentation process^9^, indicating that the self-polymerized interface of BCL10 is confined and can be targeted pharmaceutically. The structure of the CB complex showed that three major types of interfaces exist in the BCL10 filamentous structure: interstrand, or type I & II, interactions and intrastrand, type III, interactions 9, 10. Residues in α2, α3 and α4 are mainly involved in inter- and intrastrand interactions 9, 10. Considering these interface areas, we designed five BCL10 peptide inhibitor (BPI) candidates, BCL10-P1, BCL10-P2, BCL10-P3, BCL10-P4, and BCL10-P5, corresponding to BCL10 amino acid residues 30-38 (α1-α2 loop), 36-49 (α2), 49-58 (α3), 58-74 (α4), and 78-92 (α5), respectively (Figure 1A), which had the potential to compete with the filamentation interfaces of BCL10 that self-associate. To enhance their intracellular penetration capability, we fused these BPIs with a membrane translocation (MT) sequence derived from the Antennapedia homeodomain 28-30. The control peptide that contains only the MT portion was named BCL10-Ctl.

### BPIs can effectively disrupt BCL10 filament formation

To evaluate the potential effects of these BPI candidates on BCL10 filament formation, we conducted an *in vitro* filamentation assay as described previously 9. By examining the reaction products under an electronic microscope, we found that BCL10-P2 and BCL10-P4, but not BCL10-P1, BCL10-P3, BCL10-P5 or BCL10-Ctl, effectively disrupted BCL10 filament formation (Figure 1B). None of these inhibitors interfered with TEV cleavage activity (Figure S1A). Through co-immunoprecipitation assays, we confirmed that biotinylated BCL10-P2 and BCL10-P4 directly interacted with MBP-BCL10 (Figure 1C). To evaluate the impact of these BPIs on intracellular BCL10 filamentation, HeLa cells overexpressing WT BCL10 were treated with different BPIs for 24 h. Immunofluorescence staining (IF) of BCL10 was conducted to examine the formation of intracellular BCL10 filaments (Figure 1D and Figure S1B-C). Our data showed that the percentages of cells containing filamentous BCL10 were significantly reduced after treatment with BCL10-P2 and BCL10-P4 but the other BPI candidates were not affected (Figure 1E). Further analysis showed that the inhibitory effect of BCL10-P4 on BCL10 filamentation was concentration-dependent (Figure S1D-F). In BCL10-overexpressing HeLa cells, the protein level of overexpressed BCL10 was much higher than that of endogenous BCL10 in the HBL1, TMD8 or OCI-LY3 cells (Figure S1G); therefore, the concentration of BCL10-P4 required to inhibit the activity of BCL10 was much higher. Taken together, our data indicated that BCL10-P2 and BCL10-P4 are promising candidate BPIs that can effectively disrupt BCL10 filament formation in human cells.

### BPIs selectively repressed the growth of ABC-DLBCL cells

**Figure 2:**
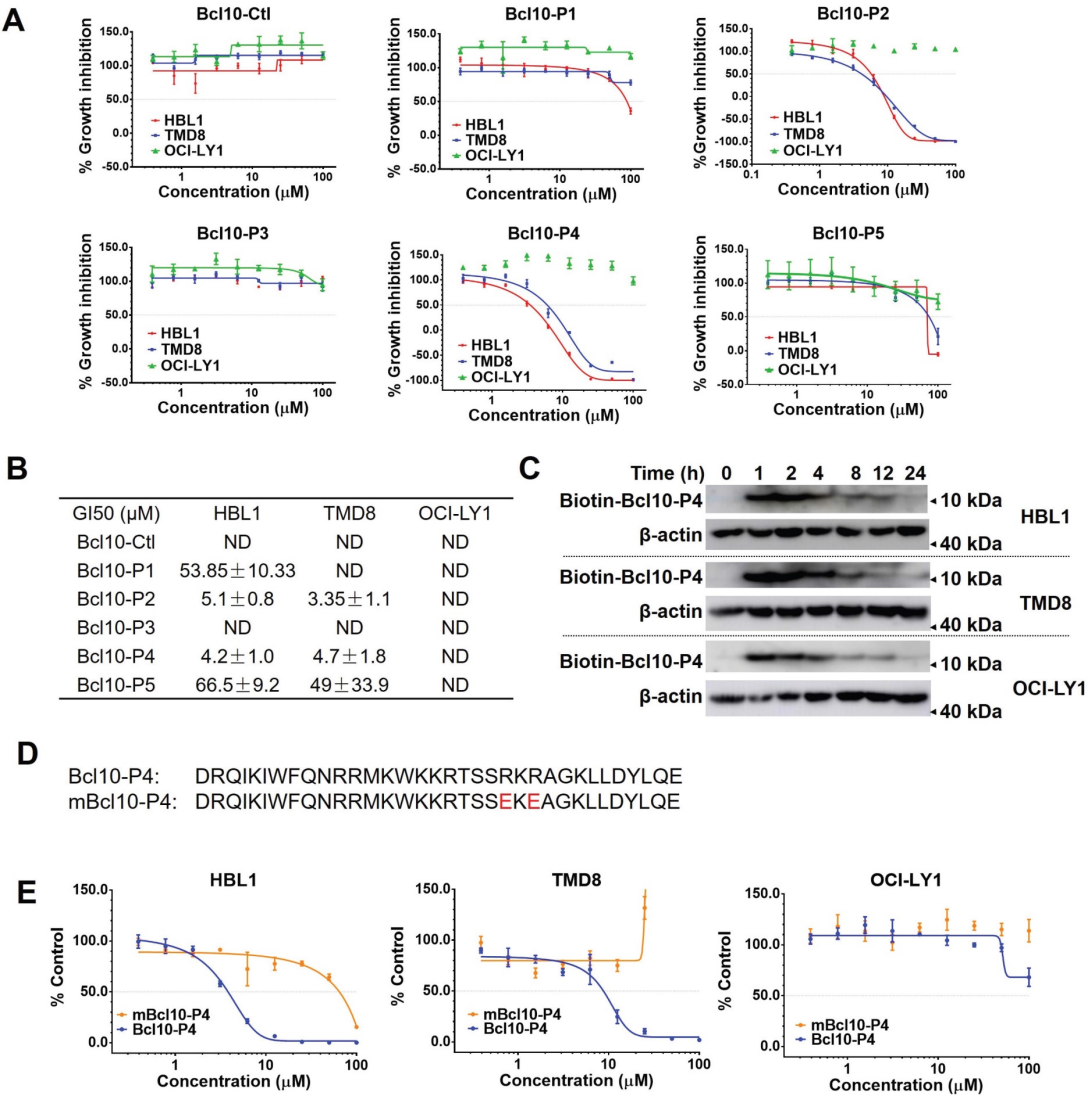
** BPIs selectively repressed the proliferation of ABC-DLBCL cells. A**. Proliferation plots of cells exposed to different BPI candidates at increasing concentrations for 72 h. The data are reported as the mean ± SD of three independent experiments. **B**. Fifty percent growth inhibition values (GI50) were calculated for the BPIs in HBL1, TMD8, and OCI-LY1 cells. ND: not determined. **C**. Pharmacological kinetics of BCL10-P4 in the HBL1, TMD8, and OCI-LY1 cells. The cells were exposed to biotinylated BCL10-P4 for the indicated times, and the presence of biotin-BCL10-P4 in the cells was blotted using streptavidin. **D**. The sequences of BCL10-P4 and mBCL10-P4 are displayed. Residues that are critical for generating the BCL10 polymerization interface were mutated, indicated in red. **E**. The effects of BCL10-P4 and mBCL10-P4 on the proliferation of HBL1, TMD8 and OCI-LY1 cells. The cells were exposed to different concentrations of BCL10-P4 or mBCL10-P4 for 72 h. The results represent the average of three independent experiments.

We next examined the effects of BPI candidates on the proliferation of DLBCL cells. ABC-DLBCL cell lines (TMD8 and HBL1 cells) and a GCB-DLBCL cell line (OCI-LY1 cells) were exposed to increasing concentrations of different BPI candidates for 72 h. Consistent with their capability to disrupt BCL10 filament formation, BCL10-P2 and BCL10-P4 displayed a strong and specific inhibitory effect on the growth of the NF-κB-addicted ABC-DLBCL cells (HBL1 and TMD8 cell lines) but not the NF-κB-independent GCB-DLBCL cells (the OCl-LY1 cell line) (Figure 2A-B). The 50% growth inhibition concentrations (GI50) of BCL10-P2 and BCL10-P4 in the HBL1 and TMD8 cells were between 3-6 µM (Figure 2A-B), which were approximately 20-fold lower than those of the GCB-DLBCL cells. A pharmacokinetics (PK) analysis confirmed that BCL10-P4 entered HBL1, TMD8 and OCl-LY1 cells with comparable efficiency (Figure 2C), suggesting that the selective inhibitory activity of BCL10-P4 on the ABC-DLBCL cells compared to its effect on the GCB-DLBCL cells was not due to differences in cellular uptake. Since BCL10-P4 peaked at approximately 1-2 h and disappeared by approximately 24 h, we re-evaluated the GI50 of these BPI candidates at 24 h, 48 h and 72 h post-treatment. Slightly lower GI50s were observed at 24 h in the BCL10-P2- and BCL10-P4-treated ABC-DLBCL cells (Figure S2A-B). Moreover, neither BCL10-P2 nor BCL10-P4 displayed a growth inhibition effect on the 293T and BPH-1 noncancerous cell lines (Figure S2C). This finding is consistent with the idea that these two BPIs are highly specific inhibitors of ABC-DLBCL cells.

Previously, it was found that amino acids R62 and R64 in BCL10 are critical for BCL10 filamentation. Mutating them into glutamic acids was sufficient to interfere with the BCL10 filamentation process 9, 10. If the BPIs act through competitive interactions with the BCL10 filamentation interface, then we would be able to disrupt the competition by mutating these two residues in the BPIs. Consistent with this idea, the R62E/R64E double mutation in BCL10-P4 (named mBCL10-P4) effectively abolished its inhibitory effect on the growth of ABC-DLBCL cells (Figure 2D-E).

### BPIs are effective inhibitors of NF-κB signalling pathway activity in ABC-DLBCL cells

**Figure 3:**
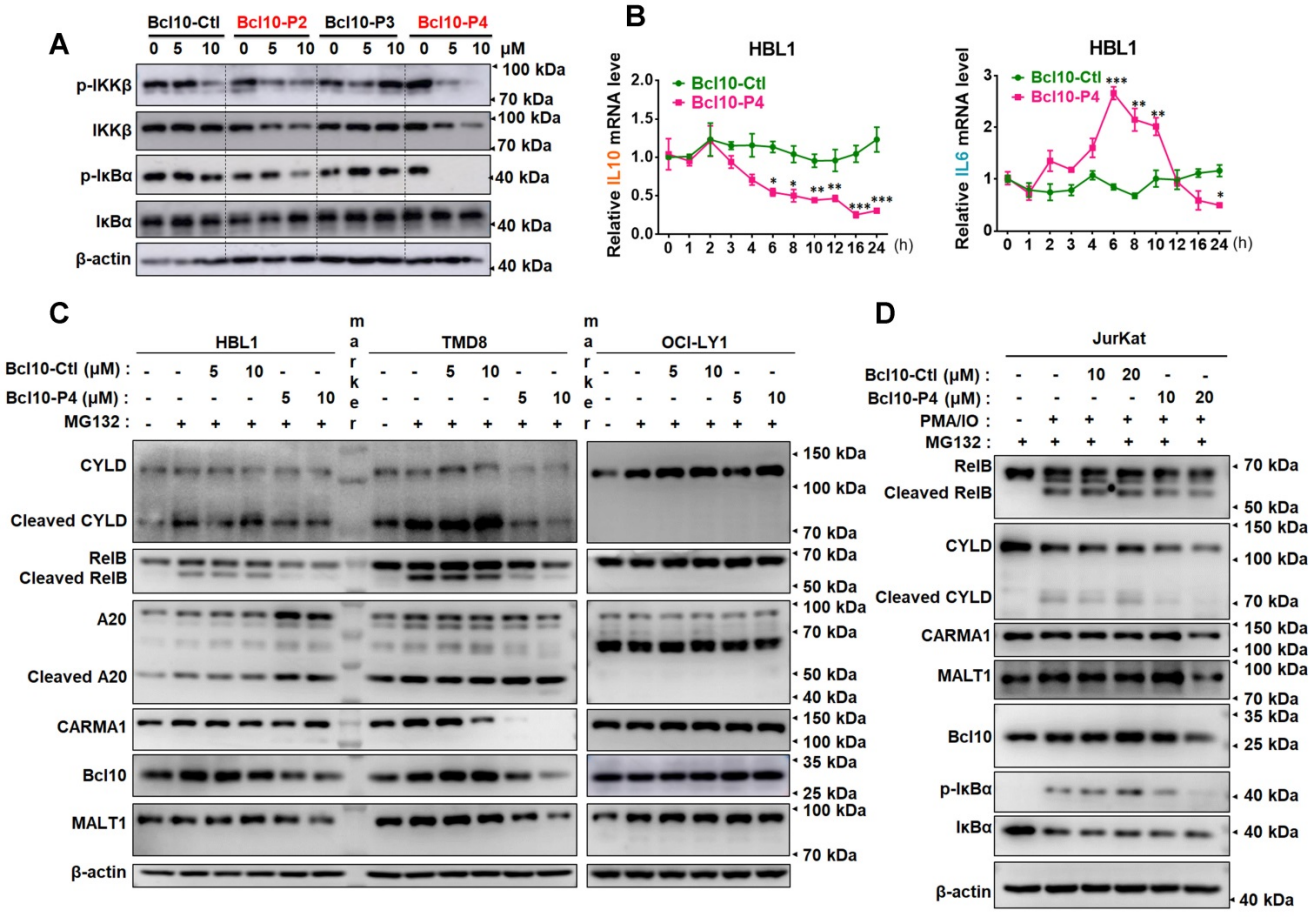
** BPIs repressed the NF-KB signalling pathway. A**. Western blot showing IKKβ, p-IKKβ, IκBα, and p-IκBα following 12 h of treatment of HBL1 cells with the indicated BPIs. **B**. Real-time quantitative PCR showing the mRNA levels of the NF-κB target genes IL-10 and IL-6 in HBL1 cells treated with BCL10-Ctl or BCL10-P4 at different time points. mRNA levels were normalized to GAPDH and are relative to time 0 point. The data are reported as the mean ± SD of three independent experiments. Statistics: one-way ANOVA with Dunnett's multiple comparison test, *p < 0.05, **p < 0.01, and ***p < 0.001. **C**. Western blot analysis of RelB, cleaved RelB, A20, cleaved A20, CARMA1, BCL10 and MALT1 in HBL1, TMD8 and OCI-LY1 cells pretreated with BCL10-Ctl or BCL10-P4 for 8 h, followed by MG132 (10 µM) treatment for 2 h. The cells were lysed in RIPA buffer with 0.1% SDS. **D**. Jurkat cells were pretreated with BCL10-Ctl or BCL10-P4 (10 µM or 20 µM) for 8 h, followed by treatment with MG132 (10 µM) for 2 h and PMA (20 ng/mL) + IO (1 µM) for 1 h. The cells were lysed in RIPA buffer with 0.1% SDS. The proteins indicated were analysed by western blotting.

We examined the effect of BPIs on the NF-κB signalling pathway in ABC-DLBCL cells. As shown in Figure 3A, BCL10-P2 and BCL10-P4, but not BCL10-Ctl or BCL10-P3, decreased the phosphorylation levels of IKKβ and IκBα and stabilized IκBα in the HBL1 cells, indicating downregulated NF-κB signalling. Similar results were also observed with the TMD8 cells but not with the OCl-LY1 cells (Figure S3A). We conducted gene expression analysis using RNA-seq of TMD8 cells that were treated with either BCL10-P4 or ibrutinib, an inhibitor of Bruton's tyrosine kinase (BTK) that blocks BCR-induced NF-κB activation. Using gene set enrichment analysis (GSEA) 31, we found that BCL10-P4 treatment of the TMD8 cells led to the significantly downregulated expression of NF-κB target genes 8, 32 after 8 h and 24 h (Figure S3B), comparable to the effect of ibrutinib treatment. RT-qPCR analysis confirmed that the expression of IL-10, a classic NF-κB target gene 33, was significantly decreased starting 6 h post-treatment of the HBL1 cells with BCL10-P4 (Figure 3B). BCL10-P4 treatment also led to decreased expression of IL-6, another NF-κB target gene, at 24 h (Figure 3B). The IL-6 mRNA level first increased and later decreased after BCL10-P4 treatment, which may be attributed to the dynamics of NF-KB signalling 34, 35. Moreover, when the HBL1 and TMD8 cells were exposed to phorbol 12-myristate 13-acetate (PMA) and ionomycin, reagents that can boost BCR-induced NF-κB pathway activation, the GI50 of BCL10-P4 in these cells increased correspondingly, as is expected of a competitive inhibitor (Figure S3C-D). Consistent with the reduced CB-SMOC function, BCL10-P4 treatment also significantly reduced the protein level of CARMA1, BCL10 and MALT1 and the enzymatic activity of MALT1, as evidenced by the decreased levels of cleaved MALT1 substrates RelB and A-20 (Figure 3C). We also investigated the effects of BPIs on Jurkat cells, as BCL10 is dispensable for their survival. The GI50 of BCL10-P4 for the Jurkat cells was higher than that for the HBL1 cells (10.28 ± 2.29 μM vs 5.1 ± 0.8 μM), as expected (Figure S3E). The Jurkat cells were stimulated with PMA/IO to activate NF-κB signalling. Treatment with BCL10-P4 led to the inactivation of NF-κB and the CBM complex, as evidenced by inhibited p-IκBα and RelB/CYLD cleavage (Figure 3D). BCL10 was also slightly degraded by BCL10-P4 treatment (Figure 3D). Therefore, BCL10-P4 treatment can inhibit the activity of the CBM complex and NF-κB signalling in PMA/IO-activated Jurkat cells. Collectively, these data suggest that BPIs are effective and specific inhibitors of CB-SMOC-mediated NF-κB pathway activation in ABC-DLBCL cells.

### BPIs can induce BCL10 protein destabilization

**Figure 4:**
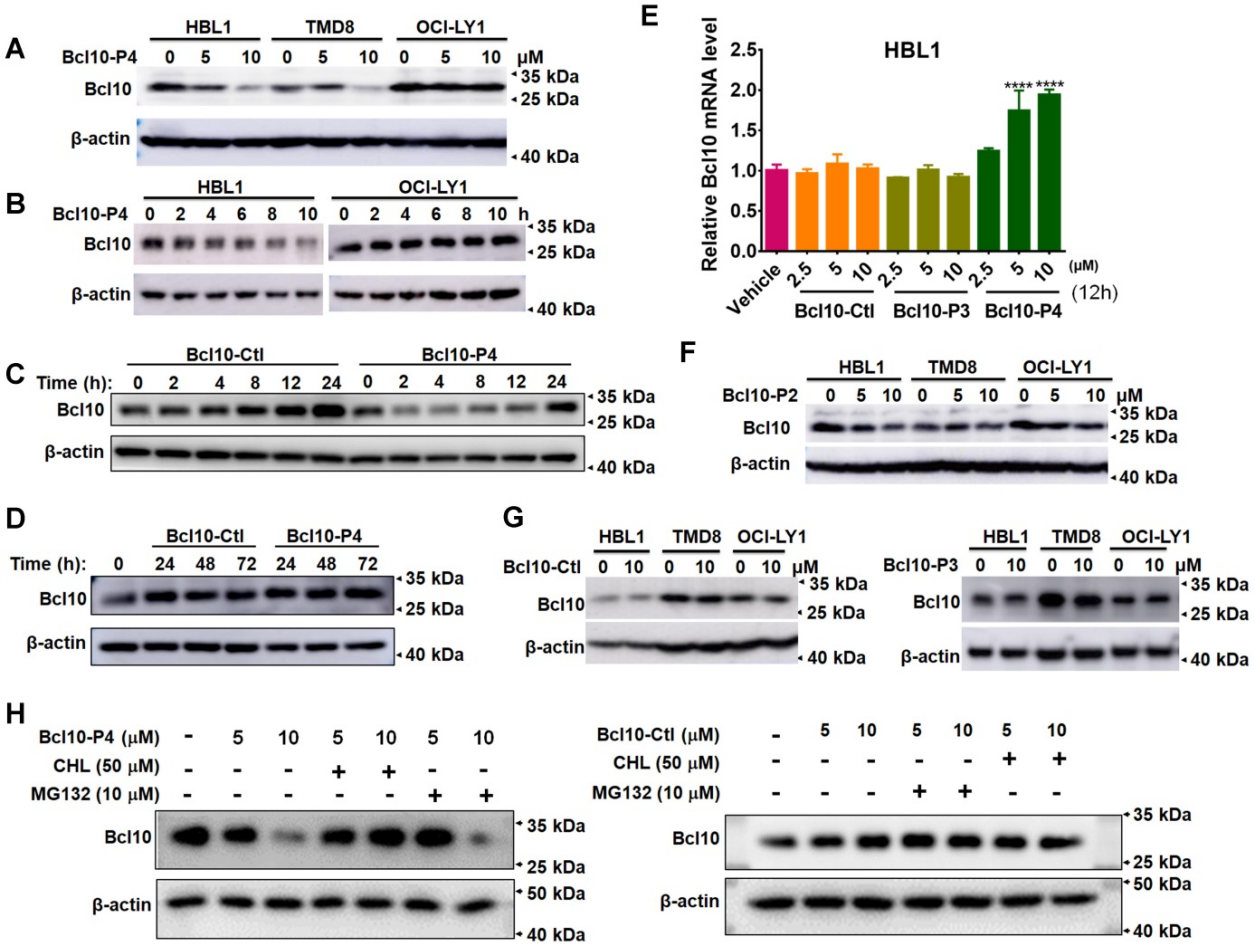
** BPIs induced BCL10 destabilization. A**. Western blot of BCL10 in cells treated with different concentrations of BCL10-P4 for 8 h. **B**. Western blot of BCL10 in HBL1 and OCI-LY1 cells treated with 5 µM BCL10-P4 for different time points. **C-D**. Western blot for Bcl10 in HBL1 cells treated with 5 µM BCL10-Ctl or BCL10-P4 for different times. **E**. Relative mRNA levels of Bcl10 in HBL1 cells treated with Bcl10-Ctl, Bcl10-P3, or Bcl10-P4 for 12 h. mRNA levels were normalized to GAPDH and relative to the vehicle-treated group. Data are the mean±SD of three independent experiments. Statistics: one-way ANOVA Dunnett's multiple comparison test. **F-G**. Western blot for Bcl10 in HBL1, TMD8 and OCI-LY1 cells treated with Bcl10-P2, Bcl10-Ctl or Bcl10-P3 for 12 h. **H**. HBL1 cells were pretreated with BCL10-P4 or BCL10-Ctl for 1 h, followed by treatment with proteasome inhibitor MG132 or autophagy inhibitor chloroquine (CHL) for 3 h. Cells were lysed in RIPA buffer with 0.1% SDS. The protein level of BCL10 was blotted.

In the aforementioned experiments, we noticed an interesting phenomenon in which the overall BCL10 protein levels seemed to be reduced in the BPI-treated cells (Figure 3D). To confirm this observation, we further investigated the impact of BPIs on BCL10 protein levels in DLBCL cells. We found that treatment with BCL10-P4 can significantly reduce BCL10 protein levels in a dose- and time-dependent manner in the HBL1 and TMD8 cells but not in the OCI-LY1 cells (Figure 4A-B). This process appeared to be rapid because the BCL10 protein level was reduced drastically, within 2 h post-treatment (Figure 4C-D). The BPI-induced BCL10 protein reduction at 2 h post-treatment with BPI was much earlier than the BPI-induced downregulation of the downstream targets of NF-κB (Figure 3B), suggesting that it is unlikely a secondary effect of NF-κB pathway inhibition. Moreover, the RT-qPCR analysis showed that treatment with BCL10-P4 but not BCL10-Ctl or BCL10-P3 led to significant upregulation of Bcl10 mRNA levels at 12 h in the HBL1 cells (Figure 4E), indicating that the BPI-induced BCL10 protein reduction was not due to reduced Bcl10 mRNA expression. This Bcl10 mRNA upregulation might account for the recovery of BCL10 protein levels approximately 24 h post-treatment with BPI (Figure 4C-D). Similar results were observed with the treatment with BCL10-P2, but not with BCL10-Ctl or BCL10-P3 (Figure 4F-G). Previous studies also reported that the activation of NF-κB triggers the gradual degradation of BCL10, reducing the impact of NF-κB signalling. Proteasome-dependent and autophagy-dependent degradation pathways have been suggested for this process 36-39. The disappearance of a western blot band is not always due to protein degradation; it can be due to protein aggregation in an insoluble fraction and thus the protein is absent in the protein lysate 3, 40. To rule out this possibility, the cells in these experiments were lysed in RIPA buffer with SDS. To investigate the degradation mechanisms of the BPIs, HBL1 cells were pretreated with BCL10-P4 or BCL10-Ctl for 1 h, followed by treatment with the proteasome inhibitor MG132 or autophagy inhibitor chloroquine (CHL) for 3 h. Treatment with CHL prevented BCL10 degradation by BCL10-P4, while MG132 negligibly prevented BCL10 degradation (Figure 4H), indicating that BCL10 was mainly degraded by BCL10-P4 through the autophagy pathway. Taken together, our data suggested that BPIs not only can disrupt BCL10 filamentation but can also induce BCL10 protein destabilization.

### BPIs selectively suppress the growth of CB-SMOC-dependent DLBCL cells and induce their apoptosis and cell cycle arrest

**Figure 5:**
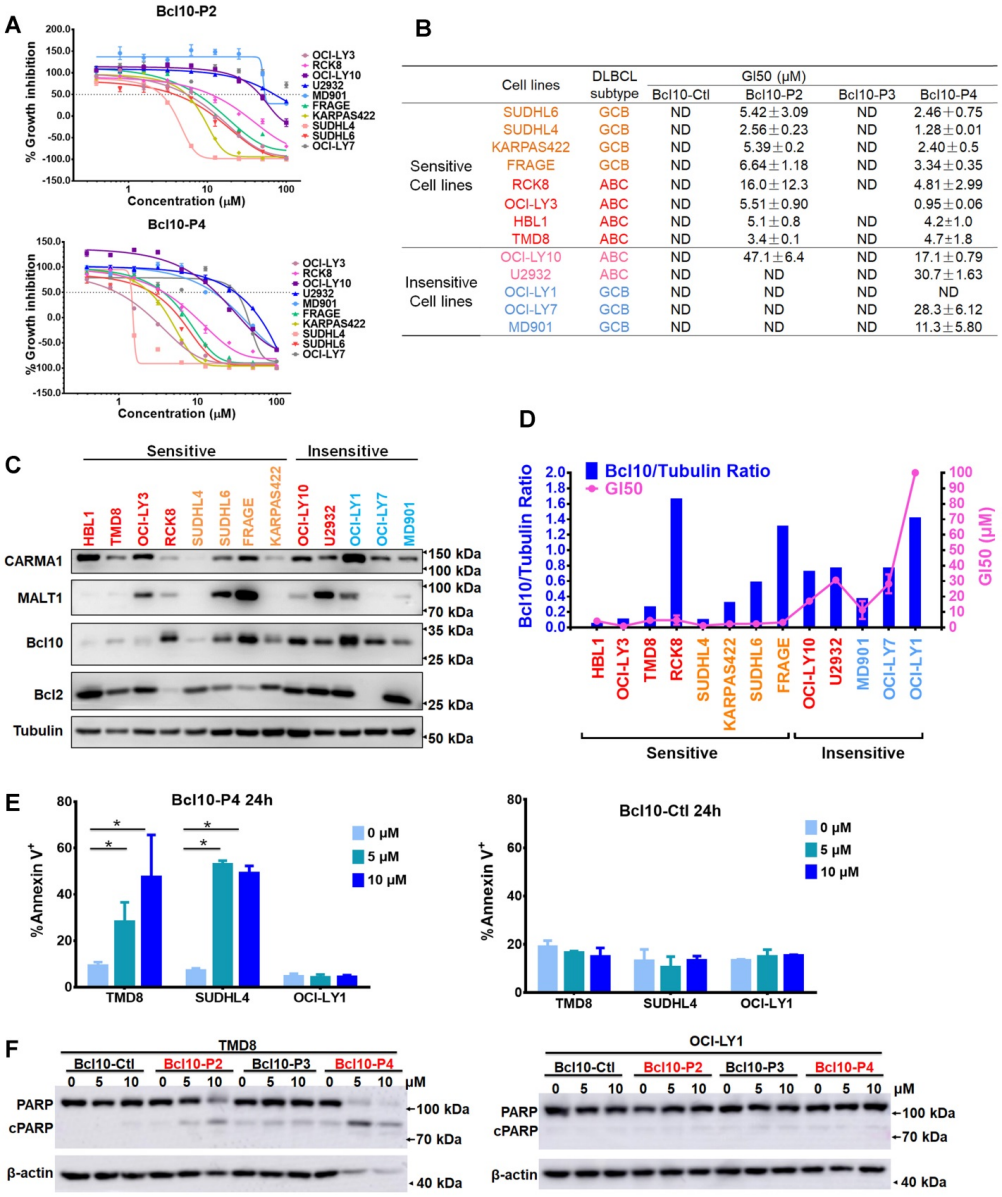
** BPIs selectively suppressed the growth of BCL10-dependent DLBCL cells. A**. Cell proliferation curves for 13 DLBCL cell lines exposed to Bcl10-P2 and Bcl10-P4 for 24 h. Experiments were performed independently in triplicate, and the data are presented as the means ± SD. **B**. GI50 values for the 13 DLBCL cell lines exposed to different BPIs. ND, not determined. **C-D**. Thirteen DLBCL cell lines were harvested and lysed in RIPA buffer. Western blot analysis showed the endogenous protein expression of CARMA1, MALT1, BCL10 and BCL2 in these cell lines. The normalization of the expression level of BCL10 to tubulin is shown in D. The GI50 of BCL10-P4 in these cell lines was also plotted.** E**. Apoptosis rate was assessed by annexin V^+^/PI^-^ and annexin V^+^/PI^+^ staining in cells treated with 5 µM BCL10-P4 or BCL10-Ctl for 24 h. The y axis shows the sum of the percentage of annexin V^+^/PI^-^ and annexin V^+^/PI^+^ cells. The data are reported as the mean±SD of two independent experiments. Statistics: one-way ANOVA with Dunnett's multiple comparison test. **F**. Western blot showing PARP and cleaved PARP from the TMD8 and OCI-LY1 cells following 12 h treatment with the indicated BPIs.

We next examined the biological effects of these BPIs on a larger panel of DLBCL cell lines (Figure 5A-B). As negative controls, BCL10-Ctl and BCL10-P3 had no significant growth inhibition effects against any of the tested DLBCL cells, even at 50 μM (Figure 5B and Figure S4A). In contrast, BCL10-P2 and BCL10-P4 exhibited strong growth inhibition effects on most ABC-DLBCL cell lines with GI50 values less than 10 μM. The only exception was U2932, which is known to harbour an activation mutation of TAK1 that is downstream of CB-SMOC 41, rendering its induction of NF-κB activation independent of the CB-SMOC. In contrast, many typical GCB-DLBCL cells, including OCI-LY1, OCI-LY7 and MD901 cells, were not sensitive to the BCL10 inhibitors, as expected (Figure 5A-B). To exclude the possibility that these cells were not sensitive to BPIs because of inefficient BPI uptake, we examined the PKs of the BPIs in these cells. The results showed that these BPI-insensitive GCB-DLBCL cells had BCL10-P4 PK profiles similar to those of the BPI-sensitive cells (Figure S4B). Surprisingly, a number of GCB-DLBCL cell lines (SU-DHL4, SU-DHL6, Karpas-422 and FRAGE cells) were also sensitive to the BPIs, suggesting that their functions are probably CB-SMOC-dependent.

To investigate the mechanisms of BPI sensitivity of DLBCL cells, we first explored whether there is a correlation between BCL10 polymerization status and sensitivity to BPIs. It was previously observed that proteins that form oligomer structures aggregate in an insoluble fraction and thus were absent in the cell lysate 3, 40. Disrupting protein oligomerization by SDS solubilizes proteins in cell lysates. Therefore, we were able to discern the polymerization status of proteins by calculating the ratio of the protein levels in the cells under these two different conditions: DLBCL cells were lysed in regular RIPA buffer (Figure 5C-D) or in RIPA buffer containing 0.1% SDS (Figure S4C-D), and the endogenous BCL10 expression levels were measured. The BCL10 polymerization index (BCL10 polymerization index=BCL10^RIPA_with_SDS^/BCL10^RIPA^) was calculated, and the results revealed that, except for RCK8 and FRAGE cells, the cells with a high BCL10 polymerization index were more sensitive to the BPIs (Figure S4E), indicating a close correlation between BCL10 polymerization state and BPI sensitivity. The OCI-LY10 ABC-DLBCL cell line was not sensitive to any BPI, which was probably due to its low BCL10 polymerization index.

We next examined whether the survival of GCB-DLBCL cells that are sensitive to BPIs is dependent on BCL10. We used shRNA to generate BCL10-knockdown (KD) cells (Figure S4F) and found that BCL10 downregulation significantly suppressed the growth of SU-DHL-4, SU-DHL-6 and Karpas-422 cells (Figure S4F). Interestingly, SU-DHL-4 cells are insensitive to MALT1 inhibition 42. Thus, their sensitivity to BPIs suggests that the direct inhibition of CB-SMOC may confer a broader antitumor effect than targeting its downstream effectors. When BCL10-silenced GBC cells were treated with BCL10-P4, only a slight change in the GI50 value was observed compared with that of the control cells (Figure S4G).

We also found that BCL10-P4 treatment led to the degradation and inactivation of the CBM complex in the sensitive cell lines but not in the insensitive cell lines (Figure S5A). To explore whether BPI specifically degrades activated BCL10, BPI-sensitive and BPI-insensitive cells were treated with BCL10-Ctl or BCL10-P4 (5 μM) for 8 h, and then were stimulated with PMA/IO for 1 h. PMA/IO treatment significantly activated the CBM complex in the ABC-DLBCL cells but mildly activated it in the GCB-DLBCL cells, as evidenced by the increase in RelB cleavage (Figure S5B). BCL10-P4 degraded BCL10 in the sensitive cells, but no significant BCL10 degradation was observed in the insensitive cells, which may be because PMA/IO only mildly activated the CBM complex in these cells (Figure S5B).

To further characterize the biological basis of BCL10-P4-induced growth inhibition in the DLBCL cells, we conducted an apoptosis analysis. We found that BCL10-P4 treatment induced significant apoptosis of the CB-SMOC-dependent DLBCL cells (TMD8 and SU-DHL4 cell lines) in a dose-dependent manner, while little apoptosis induction was observed for the CB-SMOC-independent DLBCL cells (OCI-LY1 cell line) (Figure 5E-F). As negative controls, neither BCL10-Ctl nor BCL10-P3 induced significant apoptosis in any of the aforementioned cell lines (Figure 5E-F and Figure S6A-B). The cell cycle analysis revealed that BCL10-P4 treatment resulted in an accumulation of CB-SMOC-dependent TMD8 and SU-DHL4 cells in the G0/G1 phases and their reduction in the S phase, in a dose-dependent manner, while no significant changes in the cell cycle profile were observed for the BCL10-P4-treated OCl-LY1 cells (Figure S6C). Treatment with BCL10-Ctl or BCL10-P3 did not result in cell cycle arrest in any of the aforementioned cell lines (Figure S6D-E). Taken together, our data indicated that the BPIs selectively induced apoptosis and cell cycle arrest in the CB-SMOC-dependent DLBCL cells.

### BPIs with the D-retro inverso configuration showed enhanced intracellular stability and maintained biological activity

**Figure 6:**
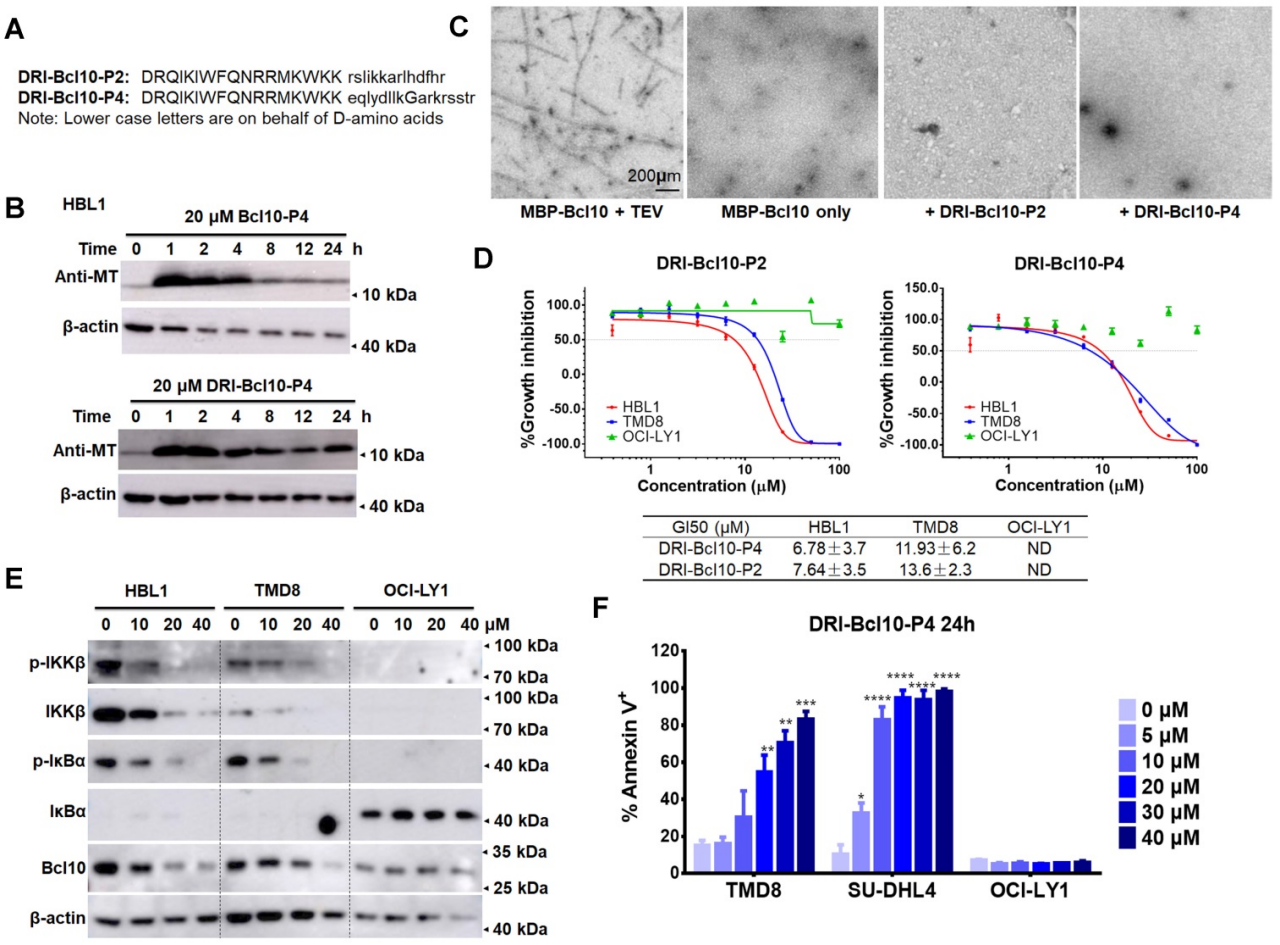
** Retro-inverso form of BPIs showed enhanced intracellular stability and maintained biological activity. A**. The sequences of the D-retro-inverso (DRI) forms of BCL10-P2 and BCL10-P4. **B**. Western blot showing BCL10 in HBL1 cells to compare the pharmacokinetics between BCL10-P4 and DRI-BCL10-P4 treatments. **C**. EM micrographs showing that DRI-BCL10-P2/P4 inhibited Bc10 filament formation *in vitro*. **D**. Dose-response curves of DRI-BCL10-P2/P4 in HBL1, TMD8 and OCI-LY1 cells. The GI50s are indicated. The experiments were performed independently in triplicate. The data are presented as the means ± SD. Statistics: one-way ANOVA with Dunnett's multiple comparison test. **E**. Western blot showing IKKβ, p-IKKβ, IκBα, and p-IκBα in the HBL1, TMD8 and OCI-LY1 cells treated with DRI-BCL10-P4 at the indicated concentrations for 12 h. **F**. Apoptosis rate was analysed by annexin V^+^/PI^-^ and annexin V^+^/PI^+^ staining of cells treated with DRI-BCL10-P4 for 24 h. The y axis shows the sum of the percentage of annexin V^+^/PI^-^ and annexin V^+^/PI^+^ cells. The data are reported as the mean ± SD of two independent experiments.

Small peptides tend to have a relatively short half-life *in vivo* due to protease degradation, presenting a major challenge for their pharmaceutical application 43, 44. Converting a natural peptide to its D-retro inverso configuration (DRI form) is a commonly used strategy to improve its resistance to protease degradation 45-47. Therefore, we synthesized DRI versions of BCL10-P2 and BCL10-P4 for further examination (Figure 6A). We first examined the PKs of BCL10-P4 or DRI-BCL10-P4 in HBL1 cells. Initially, both BCL10-P4 and DRI-BCL10-P4 were similarly taken up by the HBL1 cells and were maintained at similarly high levels within the first 4 h post-treatment. However, the intracellular level of BCL10-P4 decreased rapidly thereafter, while the level of DRI-BCL10-P4 was maintained for a much longer time (Figure 6B and Figure S7A). These data indicated that DRI-BCL10-P4 possessed enhanced intracellular stability, as expected. We then proceeded to systematically examine the biological activities of DRI-BCL10-P2/P4. Our data showed that these DRI-BPIs possessed biological functions similar to those of their normal counterparts. Specifically, DRI-BCL10-P4 interacted with BCL10 (Figure S7B), effectively disrupted BCL10 filament formation (Figure 6C and Figure S7C), selectively suppressed the growth of CB-SMOC-dependent cell lines (HBL1, TMD8 and SU-DHL4 cells) (Figure 6D and Figure S7D), repressed NF-κB pathway activation in HBL1 and TMD8 cells but not in OCI-LY1 cells (Figure 6E and Figure S7E), and induced apoptosis and cell cycle arrest of TMD8 and SU-DHL4 cells in a dose-dependent manner (Figure 6F and Figure S7F). Taken together, our data indicate that these DRI-BPIs are superior to their parental forms due to their significantly enhanced intracellular stability and unimpaired biological activity.

### DRI-BCL10-P4 suppressed the growth of BCL10-dependent DLBCL tumors *in vivo*

**Figure 7:**
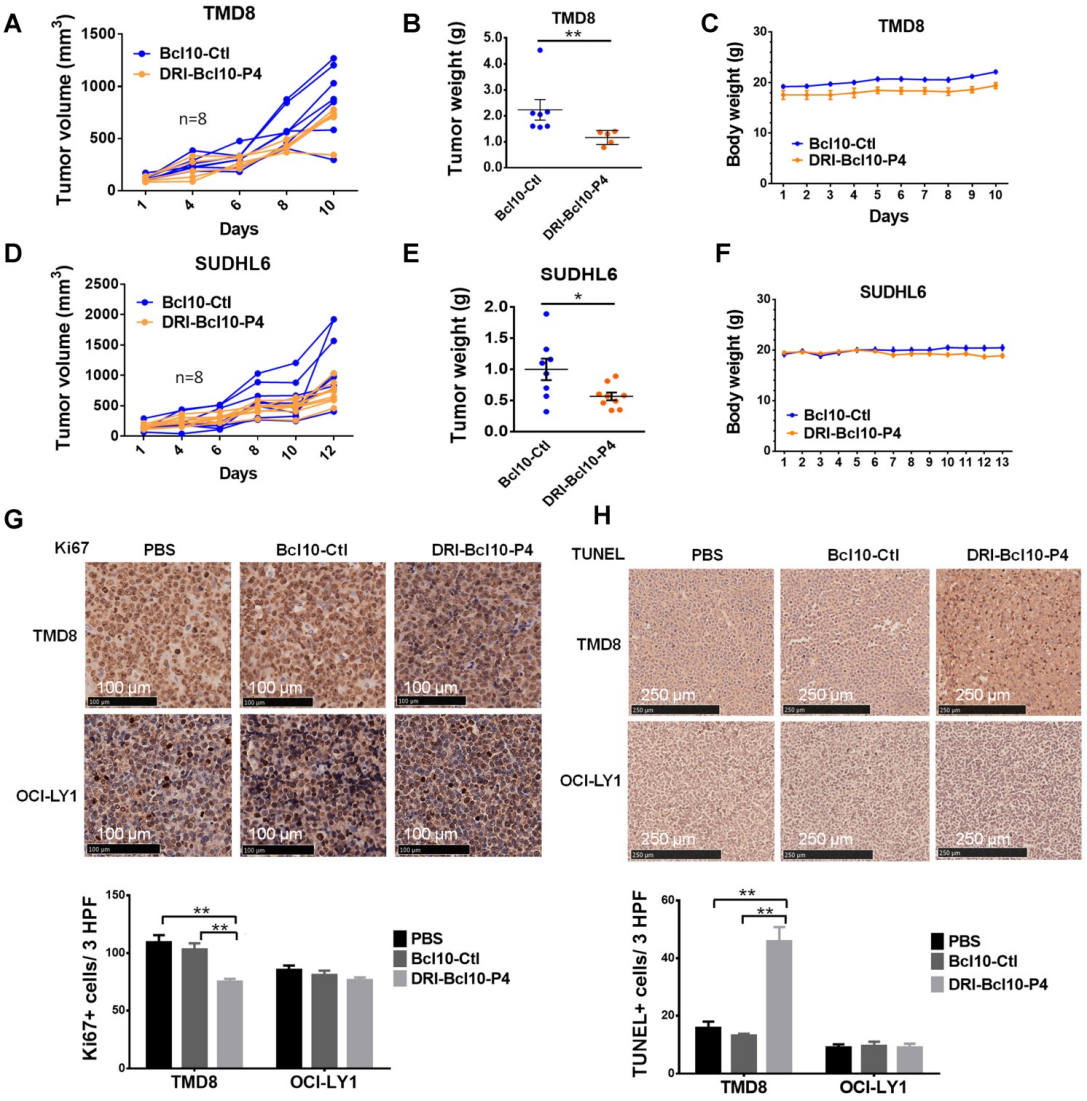
** DRI-BCL10-P4 suppressed BCL10-dependent DLBCLs *in vivo*. A-F**. Tumor growth curves for TMD8 cell (A) and SUDHL6 cell (D) xenograft tumors in the NCG mice treated with 10 mg/kg DRI-BCL10-P4 or 5 mg/kg BCL10-Ctl for the indicated days (n=8). Tumors were harvested and weighed at the end of each experiment and are shown in B-E. The body weight of the mice during the treatment are shown in C-F. **G**. Ki-67 immunohistochemical staining of BCL10-Ctl- or DRI-BCL10-P4-treated TMD8 and OCI-LY1 cell tumor sections. The quantification of Ki-67-positive cells per high-power field (HPF) is shown below. **H**. TUNEL staining in histological sections of BCL10-Ctl- or DRI-BCL10-P4-treated TMD8 and OCI-LY1 cell tumors. The quantification of the number of TUNEL-positive cells per HPF is shown below. Statistics: Mann-Whitney U test, *p < 0.05 and **p < 0.01.

Finally, we evaluated the efficacy of DRI-BCL10-P4 in xenograft mouse models. DLBCL cell lines, namely, the CB-SMOC-dependent (TMD8 and SU-DHL6 cell lines) and CB-SMOC-independent (OCI-LY1 cell lines) cells were grafted into the right flank of female NCG mice. When tumor volumes reached approximately 120-200 mm^3^, the mice were randomly selected to receive DRI-BCL10-P4 (10 mg/kg, Q.d.) or BCL10-Ctl (5 mg/kg, Q.d., to maintain a similar molar concentration) treatment daily by intravenous injection. We found that DRI-BCL10-P4 but not BCL10-Ctl significantly inhibited the growth of both TMD8 cell (*p*=0.013, Mann-Whitney U test) and SU-DHL6 cell xenograft tumors (*p*=0.049, Mann-Whitney U test) (Figure 7A-F) without inducing notable adverse effects in the mice (Figure 7C-F). On the other hand, neither peptide significantly suppressed the growth of the OCI-LY1 cell xenograft tumors (Figure S8A-C). A histological examination of the major organs confirmed that DRI-BCL10-P4 treatment did not result in discernible damage to these organs (Figure S8D). Ki67 and TUNEL staining of tumor sections revealed a significant decrease in the proliferation and a significant increase in the apoptosis rate of the DRI-BCL10-P4-treated TMD8 xenograft tumors cells compared with the DRI-BCL10-P4 effect on the control group cells. Similar effects were not observed in the OCl-LY1 cell xenograft tumors (Figure 7G-H). Taken together, our data suggest that DRI-BPIs represent an effective and safe approach to inhibit BCL10-dependent DLBCL tumor growth *in vivo*.

## Discussion

Structural and functional studies have revealed that the assembly of key signalling molecules into high-order structures or supramolecular organizing centres (SMOCs) is a common feature of signal transduction 9, 10, 48-53, which promotes proximity activation, signal amplification and transduction 14-17. CB-SMOC is such an example: it forms a high-order filamentous structure upon activation and serves as the supramolecular organizing centre for BCR/TCR-induced NF-κB activation. Bcl10 polymerizes and forms the central core of the CB-SMOC filamentous structure, which recruits and activates MALT1 and other downstream molecules in the NF-κB signalling pathway. Bcl10, specifically filamentous Bcl10, is required for the survival of ABC-DLBCL cells and other types of lymphoma cells 54, suggesting it as an attractive target for therapeutic intervention.

Marasco *et al.* generated a BCL10 inhibition peptide (corresponding to Bcl10 amino acid residues 91-98) and showed that it interfered with BCL10 self-association and repressed BCL10-induced NF-κB activation in HEK-293 cells 55. However, it was unclear whether this peptide can induce anti-tumor activity. In our study, by mimicking the BCL10-BCL10 self-polymerization interface in the CB structure model, we generated a series of novel BCL10 peptide inhibitor (BPI) candidates and identified two of them (BCL10-P2 and BCL10-P4, corresponding to Bcl10 amino acid residues 36-39 and 58-74, respectively) that effectively disrupted the BCL10 filamentation process *in vitro* and in cells. Further investigation revealed an unexpected activity of these BPIs: they can also induce a rapid destabilization of BCL10 protein in ABC-DLBCL cells. Therefore, our BPIs might act as BCL10 inhibitors through two different mechanisms. Importantly, our data showed that treatment with BPIs effectively suppressed NF-κB signalling in ACB-DLBCL cells and repressed the growth of ACB-DLBCL cells but not normal cells, implicating BCL10 targeting as a viable strategy for ACB-DLBCL therapy.

BCL10 acts upstream of MALT1; therefore, the activation of MALT1 depends on BCL10 filamentation and CB-SMOC assembly. However, BCL10 deficiency in mice was found to result in more profound phenotypes than that can be ascribed to MALT1 deficiency 21-24, suggesting a broader significance of the core CB-SMOC structure than induction of MALT1. Consistent with this result, we found that a subset of GCB-DLBCL cell lines (e.g., SU-DHL4, SU-DHL6 and Karpas-422 cells) responded to BCL10 inhibitors (Figure 5A-B) but not MALT1 inhibitors 42, suggesting a broader antitumor spectrum for BCL10 inhibition than MALT1 inhibition. Notably, MALT1 functions not only as a protease but also as a scaffold protein. MALT1 protease inhibitors only inhibited its protease function, not its scaffold function. It will be interesting to see whether inhibiting both functions of MALT1 can lead to an effect similar to that of these BCL10 inhibitors. Furthermore, a common feature of SU-DHL4, SU-DHL6 and Karpas-422 cells is that they all harbour a t(14;18) translocation, which results in elevated Bcl2 expression 56. Whether this Bcl2 upregulation renders them insensitive to MALT1 inhibition but not BCL10 inhibition remains to be investigated.

Small peptide drugs are usually not stable and prone to protease degradation in cells. We converted the peptide inhibitors into their DRI form and showed that the DRI forms of BCL10-P2/P4 not only prolonged their half-lives but also enabled them to maintain their functions. DRI-BCL10-P4 at 10 mg/kg significantly suppressed the growth of the BCL10-dependent cells (TMD8 and SU-DHL6 cell lines) but not the BCL10-independent tumor cells (OCI-LY1 cell line) in transplanted mouse models.

In addition to forming a complex with CARMA1, BCL10 also forms a complex with other CARMA family proteins, such as CARMA2, CARMA3 and CARD9, to integrate signals from multiple receptors (such as GPCR, RTK, and Dectin-1/2) to activate NF-κB 57. It has been reported that gain-of-function mutations in CARMA2 lead to spontaneous psoriasis-like skin inflammation 40, 58. Aberrant CARMA3/BCL10/MALT1 has been indicated in several cancers, including ovarian cancer, breast cancer, and prostate cancer 59-61. Therefore, the inhibition of BCL10 may represent a potentially novel therapeutic method for different CB-SMOC-dependent disease conditions, ranging from ABC-DLBCL and other aggressive cancers to inflammatory diseases such as psoriasis. Further studies are needed to test BPI applications in these disease conditions.

## Supplementary Material

Supplementary figures and tables.

## Abbreviations

ABC-DLBCLs: activated B cell-like diffuse large B-cell lymphomas
GCB-DLBCLs: Germinal centre B cell-like diffuse large B-cell lymphomas
CBM complex: CARMA1, BCL10 and MALT1 complex
CB-SMOC: CARMA1-BCL10 supramolecular organizing centre
BPIs: BCL10 peptide inhibitors
DRI: D-retro inverso configuration
BCR: B cell receptor
TLR: Toll-like receptor
CARD: caspase recruitment domain
BTK: Bruton's tyrosine kinase
PARP: poly(ADP ribose) polymerase
GSEA: gene set enrichment analysis
PMA: phorbol 12-myristate 13-acetate
MT: membrane translocation
PI: propidium iodide
IHC: immunohistochemistry
PBS: phosphate-buffered saline
R-CHOP: rituximab with cyclophosphamide, hydroxydaunorubicin, oncovin, and prednisone
RPMI: Roswell Park Memorial Institute
IMDM: Iscove's modified Dulbecco's medium
SPF: specific pathogen-free
FDA: US Food and Drug Administration
and FBS: foetal bovine serum
