# Supplementary material for: Targeting BCL10 by small peptides for the treatment of B cell lymphoma
Source: Theranostics. 2020 Sep 19;10(25):11622–36. doi: 10.7150/thno.47533 (PMC7546004; doi:10.7150/thno.47533)
Supplement: Supplementary file 1 — Supplementary figures and tables. [file thnov10p11622s1.pdf]

Supplementary data

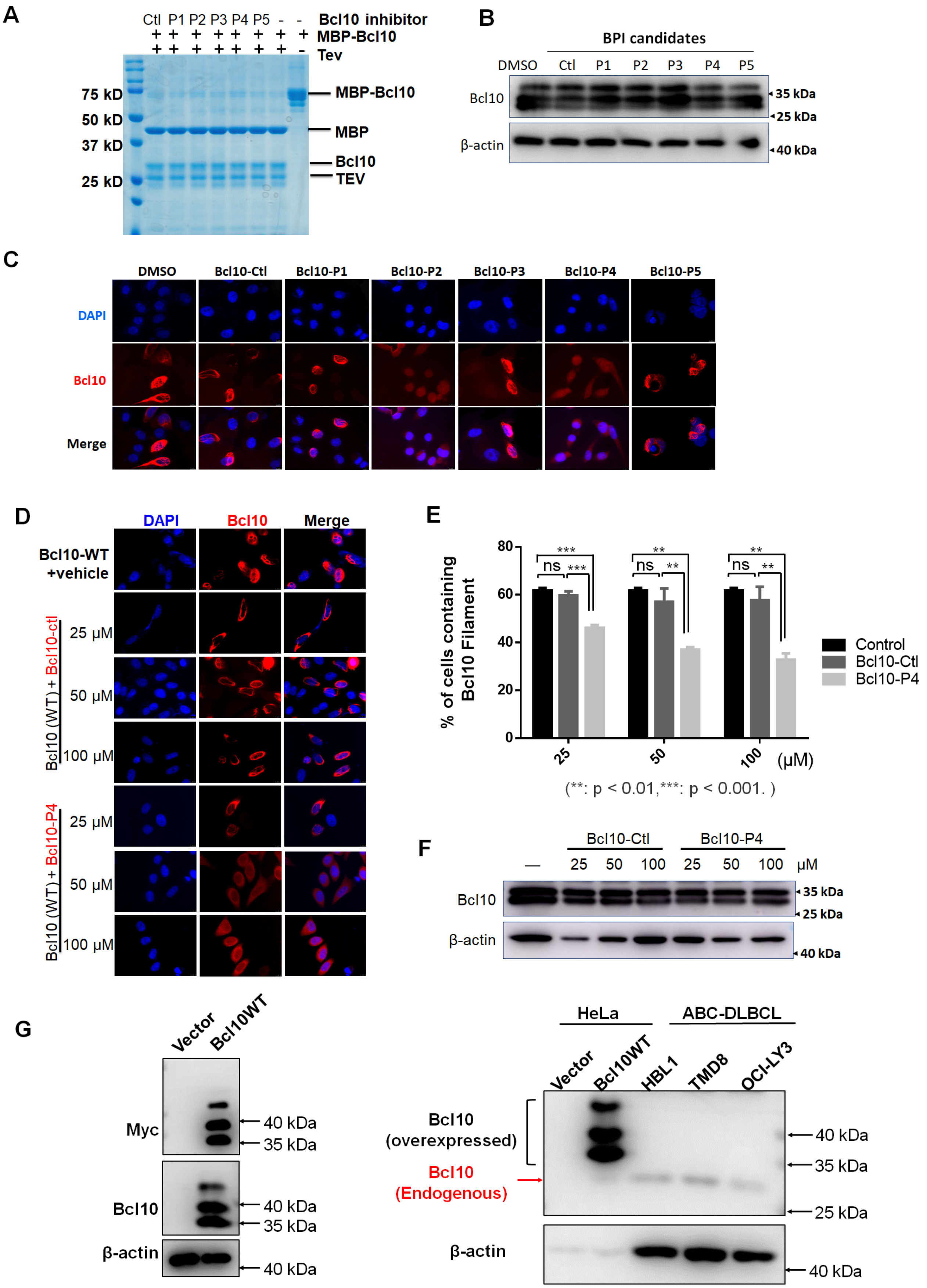

Figure S1. The effect of BPIs on Bcl10 filamentation.

A. SDS-PAGE gel showing complete cleavage of the MBP tag in all Bcl10 inhibitor treatment groups which were used for EM imaging.
B. Western blot showing overexpressed Bcl10 level in Hela cells after treatment with different BPIs.
C. Representative immunofluorescence micrographs showed the effect of BPI candidates on Bcl10 filament formation in Hela cells.

D&E. Effect of different concentrations of Bcl10-Ctl or Bcl10-P4 on Bcl10 filament formation in Hela cells. The percentage of cells containing filamentous Bcl10 were counted and shown in E.

F. Western blot showing overexpressed Bcl10 under different treatment conditions as in D.

G. Western blot of overexpressed BCL10 in Hela cells transfected with pcDNA4-myc-vector or pcDNA4-myc-Bcl10 (left). Endogenous BCL10 in Hela and DLBCL cells were also blotted (right).

A

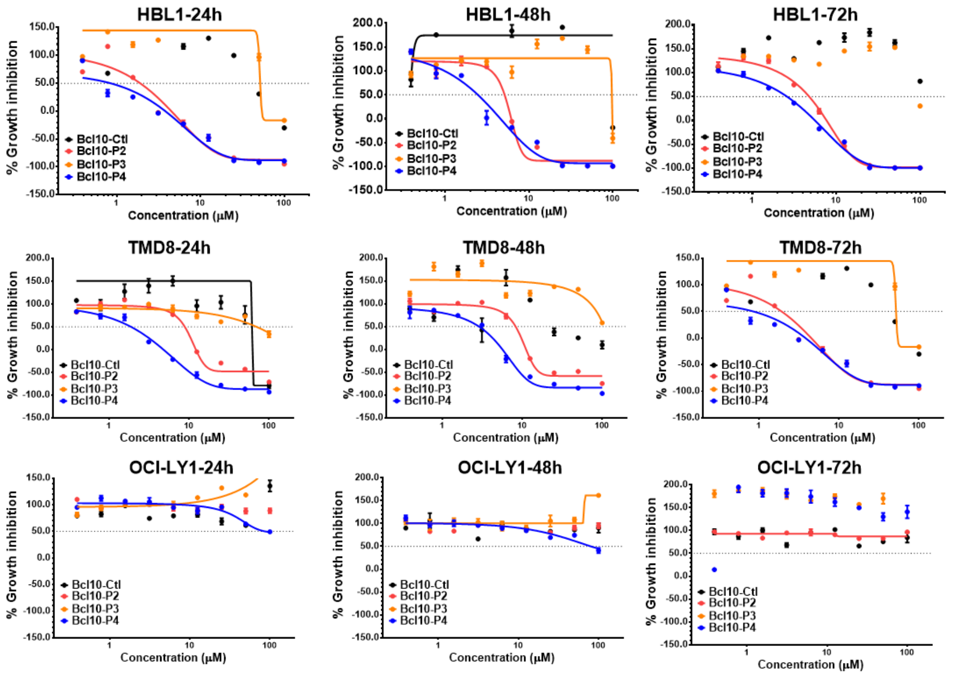

B

GI50 (μM)	HBL1			TMD8			OCI-LY1		
	24h	48h	72h	24h	48h	72h	24h	48h	72h
Bcl10-Ctl	44.3±3.25	ND	ND	>50	26.2±7.15	ND	ND	ND	ND
Bcl10-P2	1.92±0.64	4.54±0.87	4.70±0.50	7.77±1.96	8.13±1.24	8.21±2.18	ND	ND	ND
Bcl10-P3	ND	ND	ND	ND	ND	ND	ND	ND	ND
Bcl10-P4	0.658±0.23	2.51±0.53	2.56±0.23	1.78±0.87	3.11±0.72	4.25±1.09	>50	>50	ND

C

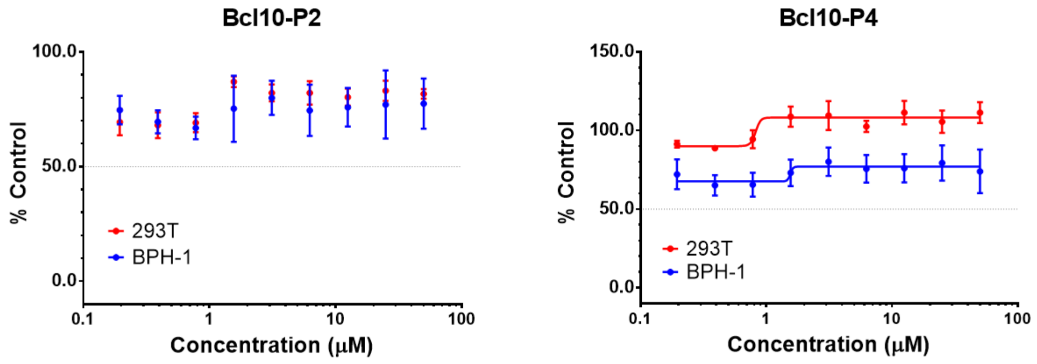

Figure S2. Bcl10-P2 and Bcl10-P4 selectively suppressed the proliferation of ABC-DLBCL cells (related to figure 2).

A. Cell proliferation curves for HBL1, TMD8 and OCI-LY1 cells exposed to different BPIs with increasing concentrations for 24h, 48h, or 72h. Data are mean±SD of three independent experiments performed.
B. 50% growth inhibition values (GI50) were calculated for BPIs in three cell lines at 24h, 48h and 72h. ND: not determined.
C. Cell proliferation curves showing the effect of Bcl10-P2 and Bcl10-P4 on normal cell line BPH1 and 293T.

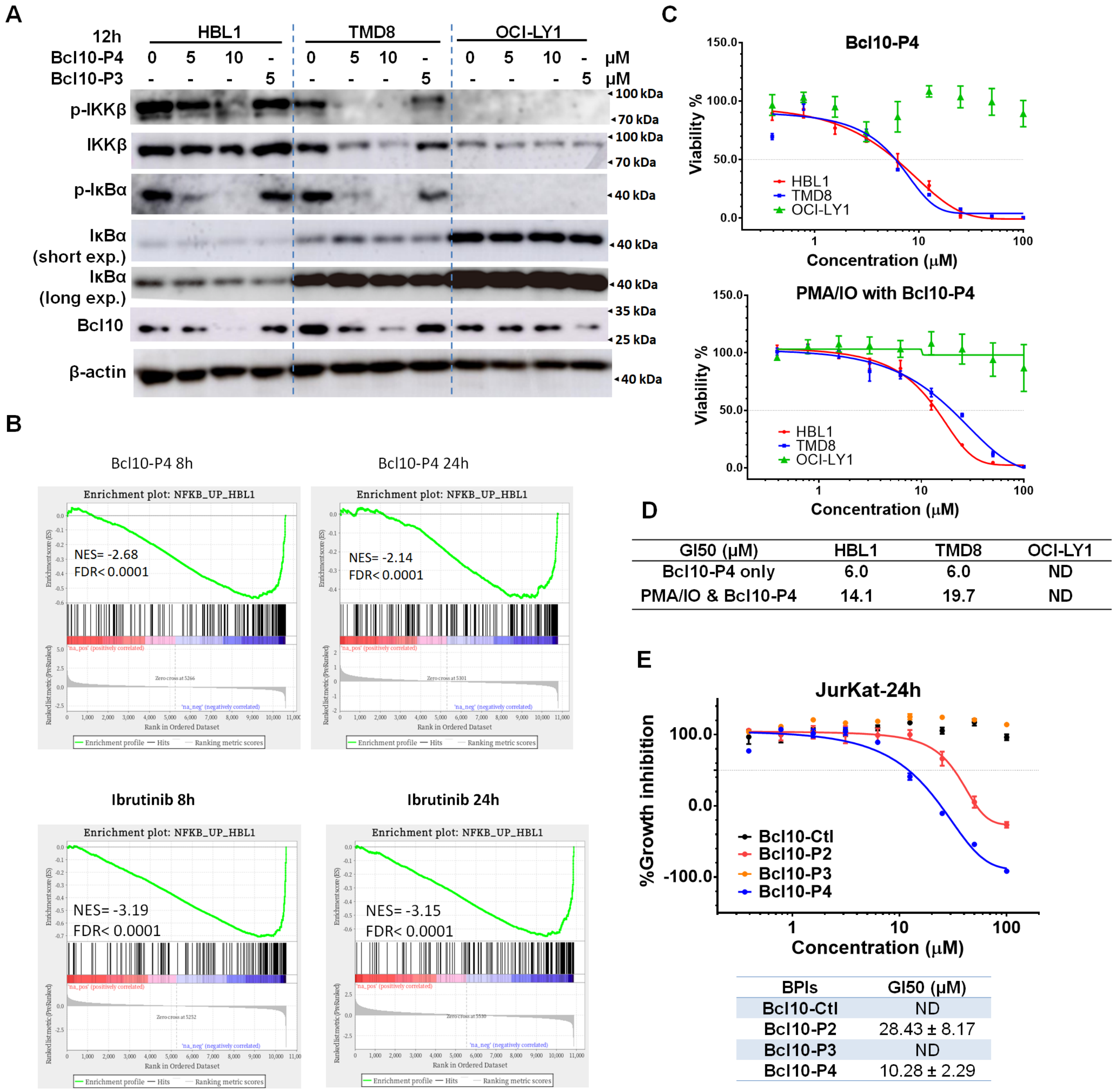

GI50 (μM)	HBL1	TMD8	OCI-LY1
Bcl10-P4 only	6.0	6.0	ND
PMA/IO & Bcl10-P4	14.1	19.7	ND

BPIs	GI50 (μM)
Bcl10-Ctl	ND
Bcl10-P2	28.43 ± 8.17
Bcl10-P3	ND
Bcl10-P4	10.28 ± 2.29

Figure S3. Effects of BPIs on NF-κB signaling.

A. Western blot for NF-κB pathway proteins IKKβ, p-IKKβ, IκBα, p-IκBα and Bcl10 following 12h treatment of BCL10-P4 or BCL10-P3 in HBL1, TMD8 or OCI-LY1 cells.

B. Gene Set Enrichment Analysis for the enrichment of NF-κB target genes (NF-κB target gene signature NF-κB_up_HBL1[54]) following BCL10-P4 treatment (5 μM) or brutinib (10 nM) I for 8h or 24h in TMD8 cells. Normalized Enrichment Score (NES) and false discovery rate (FDR) values are indicated. Experiments were performed in duplicate.

C&D. Cell proliferation curves of HBL1, TMD8 and OCI-LY1 cells pretreated with PMA/IO (20ng/mL PMA and 1ug/mL Ionomycin) or vehicle followed by treatment with Bcl10-Ctl or Bcl10-P4 for 48h. GI50 values were shown in D.

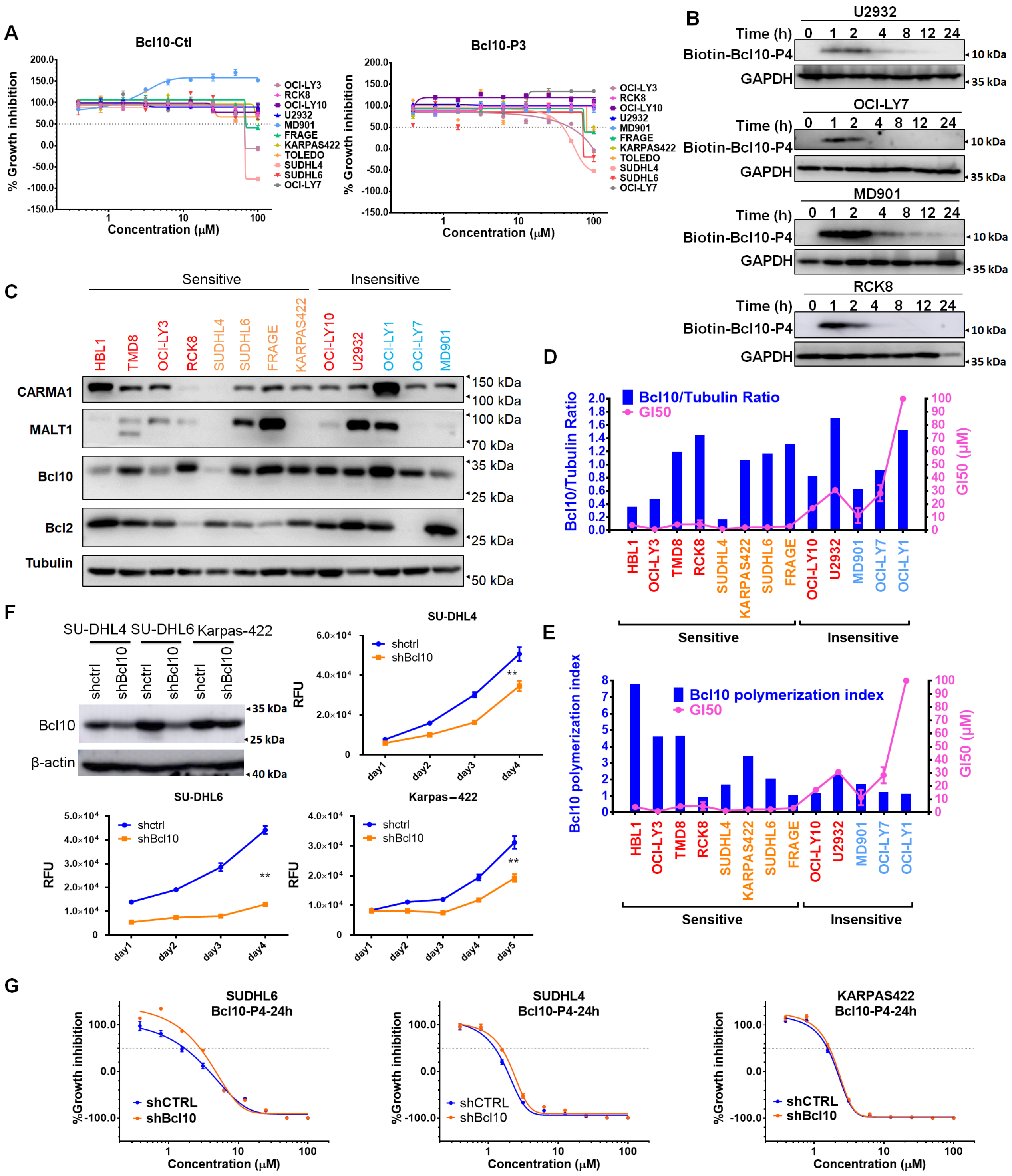

Figure S4. Effects of different BPIs different DLBCL cells and their sensitivity mechanisms.

A. Cell proliferation curves for 13 DLBCL cell lines exposed to Bcl10-Ctl and Bcl10-P3 for 24h. Experiments were performed in triplicates independently and data are mean±SD.

B. Western blot of Biotin-Bcl10-P4 in U2932, OCI-LY7, MD901, and RCK8 cells after treatment with 10μM Biotin-Bcl10-P4 for different time points as indicated.

C. 13 DLBCL cell lines were lysed in RIPA buffer with 0.1% SDS. Western blot showed the endogenous protein expression of CARMA1, MALT1, BCL10 and BCL2 in these cell lines.

D. Normalization of the expression level of BCL10 to Tubulin in S4C. The GI50 of BCL10-P4 in these cell lines were also plotted.

E. BCL10 polymerization index was calculated (BCL10 polymerization index=$BCL10^{RIPA\_with\_SDS}/BCL10^{RIPA}$) and plotted in these 13 cells lines. The GI50 of BCL10-P4 in these cell lines were also plotted.

F. Western blot analysis for BCL10 in SU-DHL4, SU-DHL6 and Karpas-422 cells transfected with shCtrl or shBCL10 lentivirus. The proliferation curves of SU-DHL4, SU-DHL6 and Karpass-422 transfected with shCtrl or shBCL10 lentivirus were shown.

G. SU-DHL4, SU-DHL6 and Karpass-422 with BCL10 knocking down were exposed to Bcl10-P4. The cell proliferation curves were shown. Experiments were performed in triplicates.

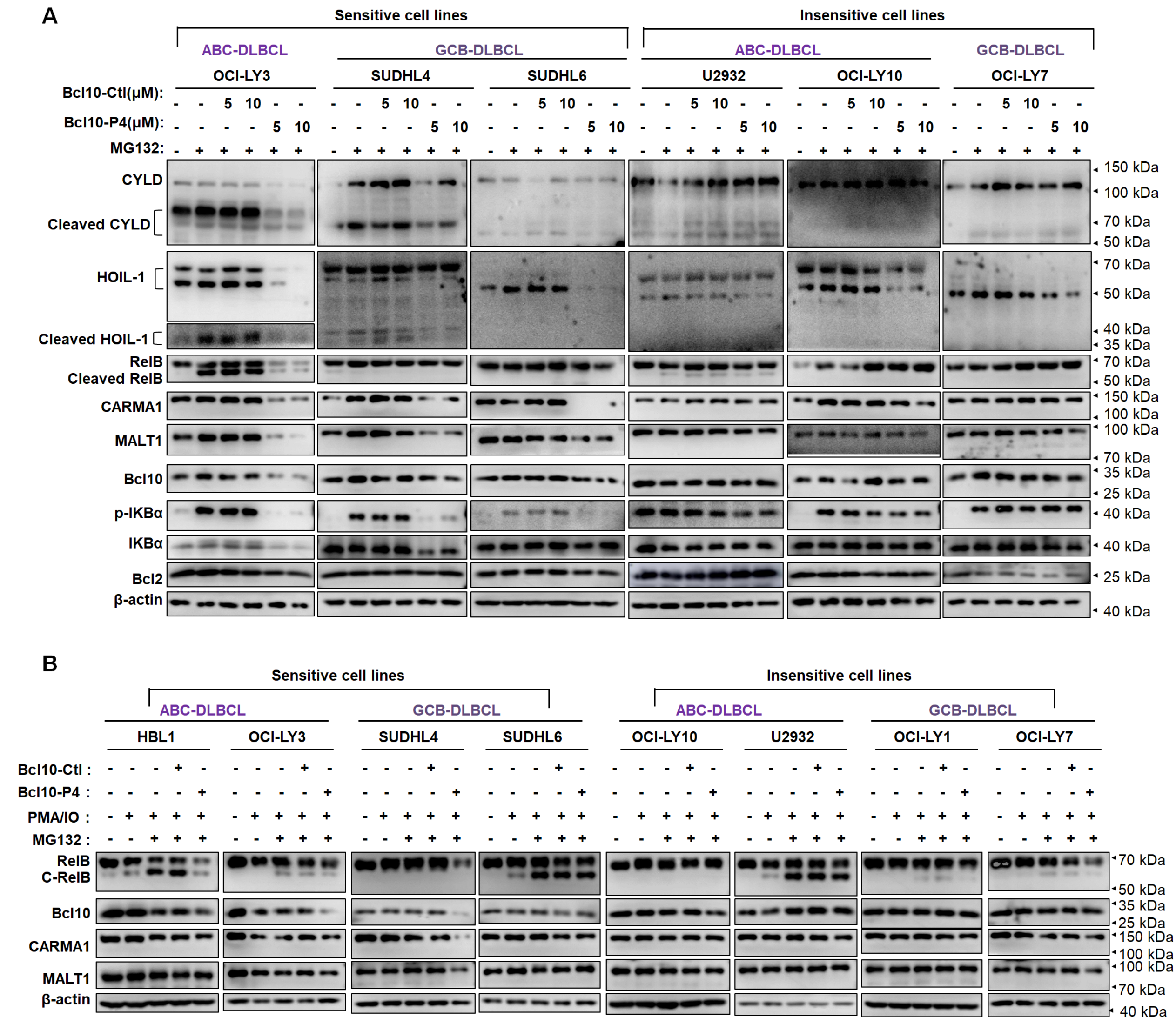

Figure S5. Effects of different BPI candidates on CBM complex and NF-KB activity.

A. BPI sensitive and insensitive cells were treated with BCL10-Ctl or BCL10-P4 (5μM) for 8 hours, followed by treatment of MG132 (10μM) for 2h. Cells were collected and lysed in RIPA buffer containing 0.1% SDS. The protein levels of indicated proteins were examined by western blot.

B. BPI sensitive and insensitive cells were treated with BCL10-Ctl or BCL10-P4 (5μM) for 8 hours,

followed by treatment of MG132 (10μM) for 2h and PMA (20ng/mL) + IO(1μM) for 1 hour. Cell were lysed in RIPA buffer containing 0.1% SDS. The protein levels of RelB, cleaved RelB (C-RelB), BCL10, CARMA1, MALT1 and β -actin were examined by western blot.

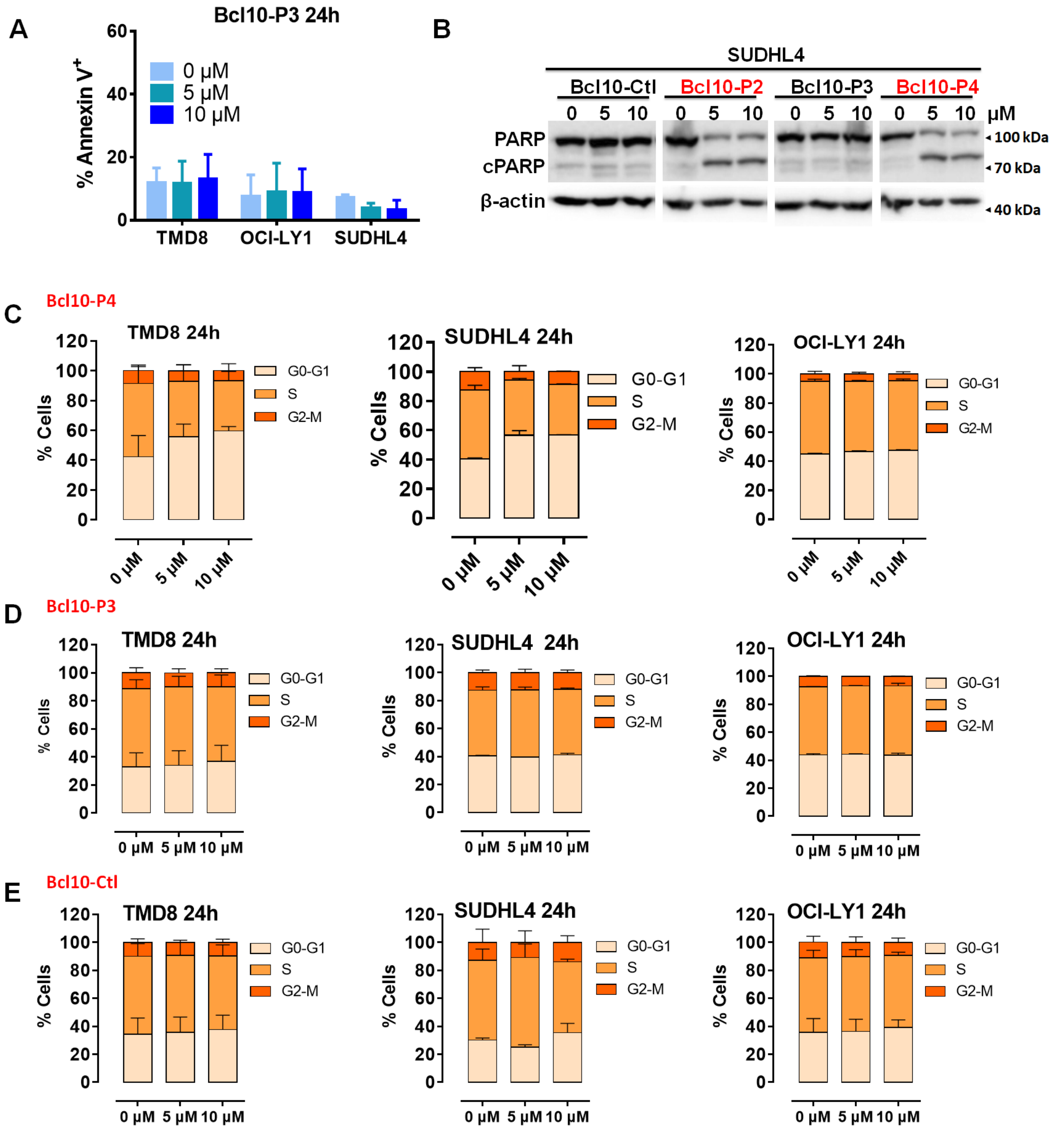

Figure S6. Effects of different BPI candidates on apoptosis and cell cycle.

A. Apoptosis was assessed by annexin V^{+}/PI^{-} and annexin V^{+}/PI^{+} staining in TMD8, OCI-LY1 and SUDHL4 cells treated with 5 μM Bcl10-P3 for 24 h. The y axis showed the sum of the percentage of annexin V^{+}/PI^{-} and annexin V^{+}/PI^{+} cells. Data are mean±SD of two independent experiments.

B. PARP cleavage were assessed by western blot in SU-DHL4 cells following 12 h treatment with indicated 5 μM BPIs.

C-E. TMD8, SU-DHL4 and OCI-L1 cells were treated with 5 μM or 10 μM BCL10-P4 (C), BCL10-P3 (D) or BCL10-Ctl (E) for 24 h, labeled with PI for 15 min, and cell-cycle distribution was analyzed by flow cytometry. Data are mean ± SD of two independent experiments.

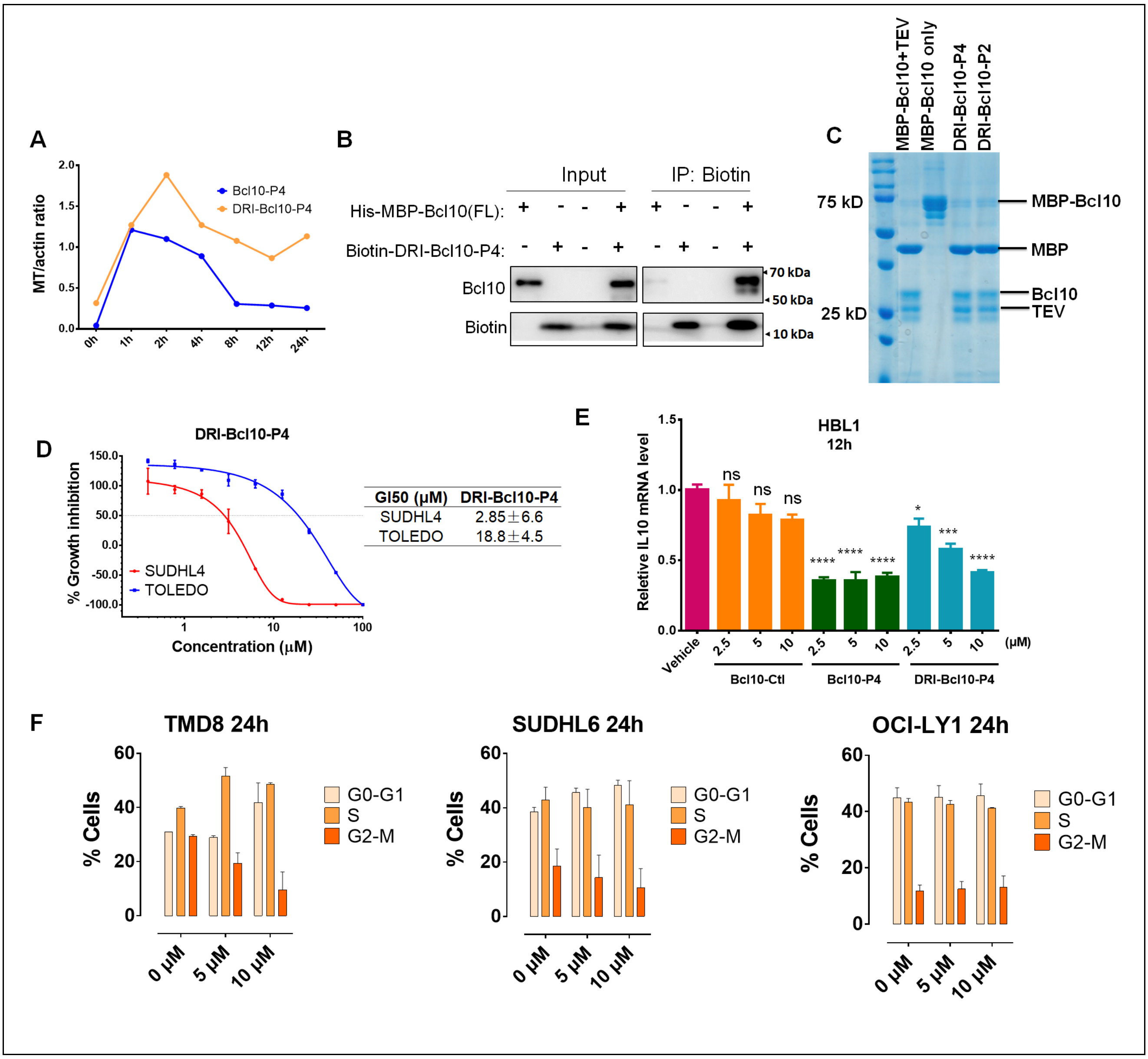

Figure S7. DRI-Bcl10-P4 retained similar biological activity as Bcl10-P4.

A. Quantification of Bcl10-P4 and DRI-Bcl10-P4 levels in HBL1 cells at different time points as in figure 6B.

B. Western blot showing co-immunoprecipitation of DRI-Bcl10-P4 with MBP-Bcl10.

C. SDS-PAGE gel showing the complete removal of the MBP tag after the treatment of DRI-Bcl10-P2 and P4 (related to figure 6C).

D. Cell proliferation plots of DRI-Bcl10-P4 in SU-DHL4 and Toledo cells. The GI50s were indicated.

E. Relative mRNA levels of IL-10 in HBL1 cells treated with Bcl10-Ctl, Bcl10-P4 and DRI-Bcl10-P4,

respectively for 12 h. mRNA levels were normalized to GAPDH and relative to vehicle treated group. The expression of IL-10 mRNA in each treatment group were compared to the vehicle group.

F. Cell cycle were analyzed by flow cytometry for TMD8, SU-DHL4, and OCI-LY1 cells that were treated with DRI-Bcl10-P4 for 24 h. Data are mean±SD of two independent experiments.

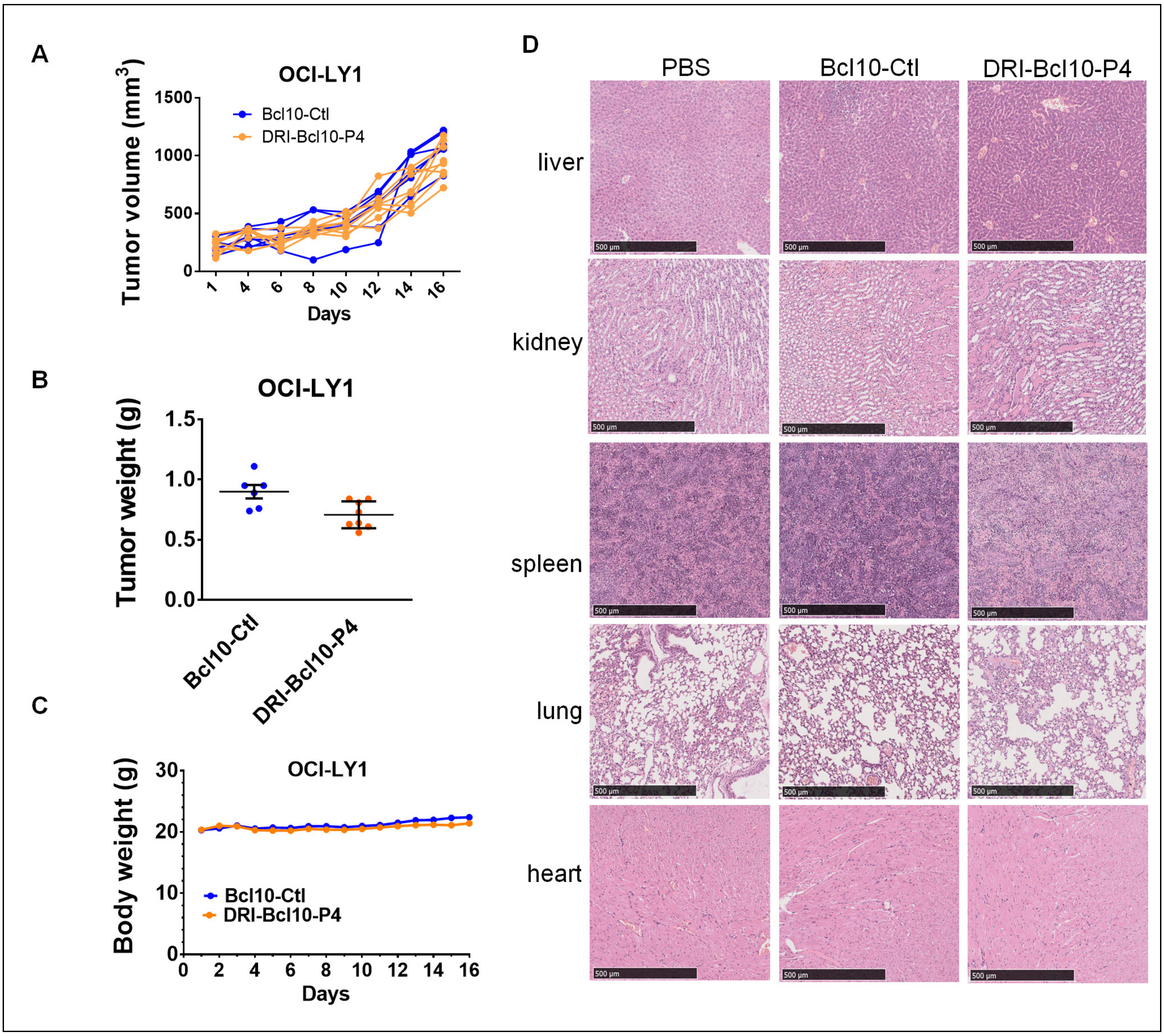

Figure S8. The effect of DRI-Bcl10-P4 in xenograft OCI-LY1 tumors.

A-C. Tumor growth curves for OCI-LY1 (A) xenografts in NCG mice treated with 10 mg/kg of DRI-Bcl10-P4 or 5 mg/kg Bcl10-Ctl for indicated days. Tumors were harvested and weighted at the end of each experiments and showed in B. The body weight of mice during the treatment were shown in C.

D. H&E staining for the indicated tissues in animals treated with PBS, Bcl10-Ctl 5 mg/kg, DRI-Bcl10-P4 10 mg/kg daily for 11 days.

Methods

Co-immunoprecipitation

MBP-Bcl10 was expressed and purified as previously described {Qiao, 2013 #85}. Purified MBP-Bcl10 was first incubated with biotinylated Bcl10-P4 in IP Buffer (20mM Tris-HClpH7.5, 300 mM NaCl, 1% NP-40) at 4 °C for 1 hour, followed by addition of 5% BSA blocked Streptavidin (SAV) MagBeads, and incubated at 4 °C for another 1 hour to immunoprecipitate biotinylated Bcl10-P4. Immunoprecipitant were washed 5 times with IP buffer and separated by SDS-PAGE. Co-immunoprecipitated MBP-Bcl10 was examined by immunoblotting using anti-Bcl10 antibody (Santa Cruz, cat. sc-5611), biotinylated Bcl10-P4 was blotted by Streptavidin-HRP (cell signal technology, 3999S).

Cell proliferation assay

shBcl10 plasmid for shRNA expression was constructed in pLKO.1 plasmid (a gift from professor Qiurong Ding). Sequences for control and target specific shRNA are as following: Control shRNA (CAACAAGATGAAGAGCACCAA); shBcl10 (GGACACCCTTGTTGAATCTAT). Lentivirus production using 293T cells with packaging plasmid pPAX2 and pMD2.G (gifts from Professor Qiurong Ding). Su-DHL4, Su-DHL6 and Karpas422 cell lines were transfected with those lentivirus respectively and selected with puromycin (Invitrogen, A1113803) for 2 weeks. Log phase growing cells were seeded at $0.25*10^6$/ml in flask and cultured as normal. The number cells were measured by flow cytometry every 24 hours and plotted as a function of time.

Real-Time Quantitative PCR (RT-qPCR)

Cells were harvested and RNA was extracted and quantified. Synthesis of cDNA was performed with DNA-free RNA samples by reverse transcription following the manufacturer’s protocol (Takara, cat. RR036A). Real time PCR was performed using SYBR Green mix (Vazyme; Cat.Q711) on a QuantStudio 6 Flex (Applied Biosystems). RNA was normalized by β-Actin or GAPDH mRNA. The following quantitative PCR primers were used:

hGAPDH-F: 5’-TCAACGACCACTTTGTCAAGC-3’
hGAPDH-R: 5’-TACTTTATTGATGGTACATGACAAGG-3’
hActin-F: 5’-CGCGAGAAGATGCCCAGATC-3’
hActin-R: 5’-TCACCGGAGTCCATCACGA-3’
hIL10-F: 5’-GGTTGCCAAGCCTTGTCTGA-3’
hIL10-R: 5’-AGGGAGTTCACATGCGCCT-3’
hIL6-F: 5’- ACTCACCTCTTCAGAACGAATTG-3’
hIL6-R: 5’- CCATCTTTGGAAGGTTCAGGTTG-3’.
hBcl10-F: 5’-GTGAAGAAGGACGCCTTAGAAA-3’
hBcl10-R: 5’-TCAACAAGGGTGTCCAGACCT-3’

Pharmacokinetics

$1x10^5$ HBL1, TMD8, OCl-LY1, OCl-LY1, U2932, MD901 or RCK8 cells were seeded in 12-well plate and exposed to 5μM biotinylated BCL10-P4 for 1h, 2h, 4h, 8h, 12h, or 24h. Cells were harvested, washed and lysed with RIPA buffer. Biotinylated BCL10-P4 in cells were determined by immunoblotting with Streptavidin (Cell signaling, cat. #3999S).

Apoptosis and cell cycle:

For apoptosis assessment, cells were treated with 5μM BPIs for 24 hours or 48 hours. Then cells were collected, washed twice with cold PBS, re-suspended in Binding Buffer and stained with AnnexinV-FITC (KeyGen Biotech, KGA703) and propidium iodide (KeyGen Biotech, KGA703) at

1:100 dilution for 15 min at room temperature in the dark. Signals from FL1 and FL3 channels were collected by MACSQuant analyzer and data were analyzed using FloJo (Ashland, OR). Early apoptosis was defined as AnnexinV+/PI-, and late apoptosis as AnnexinV+/PI+. Percentage of apoptotic cells were the sum of early and late apoptotic cells.

For cell cycle analysis, cells were washed twice with cold PBS and fixed in 70% ethanol overnight at 4°C. Fixed cells were collected at 1800 rpm for 10 min and stained with PBS containing PI and DNase-free RNase A (Sigma-Aldrich) for 1h at room temperature in the dark. DNA content was measured by BD LSRII flow cytometer and data were analyzed in FloJo.

Gene expression profiling

TMD8 or OCl-LY1 cells were treated with 5 µM Bcl10-P4, 10 nM ibrutinib or vehicle for 2h, 8h, or 24 h. Cells were harvested and RNA was extracted using TRIzol (Takara) and RNeasy kit (Tiangen). All extracts were treated with DNase in column during RNA purification. RNA integrity was determined on an Agilent 2100 Bioanalyzer (Agilent Technologies, SantaClara, CA). RNA sequencing was performed using HiseqXten-PE150 by NovogeneCo. Ltd (Beijing, China).

Gene Set Enrichment Analysis (GSEA)

RNAseq raw data are analyzed with the Salmon software [30] using default settings. Log2Fold changes and P values of differentiation gene expression between different samples are calculated using the Bioconductor - DESeq2 R package [31] using default settings. Unsupervised Hierarchical clustering analysis was conducted using the Cluster software [32]. Gene set enrichment analysis (GSEA) was conducted using Pre-ranked model of GSEA software[33,34] using default settings. Pre-defined gene sets are either extracted from the Staudt lab's signature genes database (https://lymphochip.nih.gov/cgi-bin/signaturedb/signatureDB_DisplayGenes.cgi?signatureID=276) [35]or the Molecular Signatures Database (MSigDB) [34].

Mouse xenograft experiments:

Six-week-old female $NOD^{prkdc-/-\ IL-2Rg-/-}$(NCG) mice purchased from GemPharmatech Co., Ltd were raised in a SPF-level Laboratory Animal Room. Low-passage $5x10^6$ human TMD8, SU-DHL-6 or $1x10^7$ OCl-LY1 cells at 100µl were premixed with 100µl Matrigel (Corning Biocoat, cat. # 354234) and inoculated subcutaneously into the right flank of mice. When tumors reached an average size of 150 mm^3, mice were randomly assigned into treatment groups (10 mg/kg DRI-BCL10-P4) or control groups (5 mg/kg BCL10-Ctl). Peptides were reconstituted in 5% DMSO/PBS and administered by i.v. injection. Tumor volumes were monitored by a digital caliper every other day and calculated using the formula: (smallest $diameter^2$ x largest diameter)/2. Mice were weighed every day and sacrificed at the end of the treatment. Tumors and main organs were harvested and weighted at the moment of death. All animal experiments were approved by the Animal Institute Committee of Changhai Hospital, the Second Military Medical University.

Immunohistochemistry (IHC)

Formalin-fixed paraffin-embedded tumor xenografts were sectioned, dewaxed and submitted to antigen retrieval. The anti-Ki67 (proteintech, 27309-1) antibody was used at a 1:500 dilution. Biotin-conjugated secondary antibodies (MXB biotechnologies, KIT-9710) were used. Then avidin/biotin peroxidase (MXB biotechnologies, KIT-9710) was applied to the slides. Color was developed with diaminobenzoate chromogen peroxidase substrate (MXB biotechnologies, MAX007™) and counterstained with DAB detection kit (MXB biotechnologies, MAX007™). Pictures were obtained using NanoZoomer-S60 microscope (HAMAMATSU).

TUNEL

Terminal deoxynucleotidyl transferase dUTP nick end labeling, TUNEL assay was used to detect apoptotic DNA fragmentation. All the experiments were performed according to the kit (KeyGEN Biotech, KGA703) instructions. Briefly, formalin-fixed paraffin-embedded xenografted tumors were deparaffinized and pre-treated with trypsin to expose DNA. Endogenous peroxidase was quenched using 3% hydrogen peroxide followed by incubation with TdT enzyme for 1 hour. Then, antidigoxigenin-peroxidase was applied to the slides. Color was developed with diaminobenzoate chromogen peroxidase substrate and counterstained with methyl green. Pictures were obtained using NanoZoomer-S60 microscope (HAMAMATSU).
